# Study of the Adsorption Behavior of Surfactants on Carbonate Surface by Experiment and Molecular Dynamics Simulation

**DOI:** 10.3389/fchem.2022.847986

**Published:** 2022-04-07

**Authors:** Jinjian Hou, Shuanglong Lin, Jinze Du, Hong Sui

**Affiliations:** ^1^ School of Chemical Engineering and Technology, Tianjin University, Tianjin, China; ^2^ National Engineering Research Centre of Distillation Technology, Tianjin, China; ^3^ Collaborative Innovation Center of Chemical Science and Engineering, Tianjin, China; ^4^ School of Chemical Engineering, Shijiazhuang University, Shijiazhuang, China

**Keywords:** surfactants, adsorption isotherm, wettability, molecular dynamics simulation, nanoparticles

## Abstract

Surfactants adsorption onto carbonate reservoirs would cause surfactants concentrations decrease in surfactant flooding, which would decrease surfactant efficiency in practical applications of enhanced oil recovery (EOR) processes. Different surfactants could be classified as cationic surfactants, anionic surfactants, non-ionic surfactants according to the main charge, or be classified as chemical surfactant and bio-surfactant according to the surfactant origin. However, the research on different type surfactants adsorption on carbonate reservoirs surface differences was few. Therefore, five representative surfactants (CTAB, SDS, TX-100, sophorolipid, rhamonilipid) adsorption effect onto carbonate reservoirs surface was studied. Owing to the fact that the salinity and temperature in underground carbonate reservoirs were high during the EOR process, it is vital to study the salinity effect and temperature effect on surfactant adsorption. In this study, different surfactants species, temperature and salinity adsorption onto carbonate reservoirs were studied. The adsorption isotherms were fitted by Langmuir, Freundlich, Temkin and Linear models, and the first three models fitting effect were good. The results showed that cationic surfactants adsorption quantity was higher than anionic surfactants, and the non-ionic surfactants adsorption quantity was the lowest. When the temperature increased, the surfactants adsorption would decrease, because the adsorption process was exothermic process, and increasing temperature would inhibit the adsorption. The higher salinity would increase surfactants adsorption because higher salinity could compress electric double layer. In order to decrease surfactants adsorption, SiO_2_ nanoparticles and TiO_2_ nanoparticles were added to surfactants solutions, and then surfactants could adsorb onto nanoparticles surface, then the steric hindrance between surfactant molecules would increase, which could decrease surfactants adsorption. Contact angle results indicated that surfactants adsorption made the carbonate reservoir wettability alteration. In the end, surfactants (with or without SiO_2_ nanoparticles) adsorption onto carbonate reservoirs mechanism were studied by molecular dynamics simulation. The simulation results indicated that the surfactants molecules could adsorb onto SiO_2_ nanoparticles surface, and then the surfactants adsorption quantity onto carbonate rocks would decrease, which was in accordance with the experiments results.

## 1 Introduction

In recent years, enhanced oil recovery technology has attracted extensive attention,surfactants have been widely used in the enhanced oil recovery process (Kumar et al., 2017; Saxena et al., 2017). The surfactants could decrease oil/water interfacial tension, alter the rocks surface wettability from oil-wet to water-wet, overcome capillary force, so as to improve the crude oil recovery (Kumar et al., 2008; Song et al., 2013; Das et al., 2018; Nikseresht et al., 2020). However, the loss of surfactant at the rock-water interface would decrease surfactants concentration, then decrease the surfactants efficiency of EOR process (Tangparitkul et al., 2018). The loss of surfactant can occur from the adsorption of surfactant onto reservoir surfaces (Liu et al., 2021). In addition, surfactants adsorption would influence solid surface wettability, and alter the surface property (Altamash et al., 2021; Hou and Sun 2021). Therefore, it is necessary for us to study the mechanism of surfactants adsorption on the rocks surface (Kania et al., 2021; Yusuf et al., 2021).

In recent years, the behaviors of surfactants adsorption on the minerals surface have been widely studied (Amirianshoja et al., 2013; Kumar and Mandal 2019). In recent years, many researchers focused on the carbonate rocks enhanced oil recovery, and the ions concentrations and species would influence the carbonate rocks wettability, and then influence the oil recovery (Koleini et al., 2019a; Dehaghani and Badizad 2019). Amit Kumar et al.(Kumar and Mandal 2019) studied the adsorption behavior of the zwitterionic surfactant on the sandstone and carbonate surface, and the results showed when the salinity increased, the surfactants adsorption quantity would increase, and the adsorption would alter sandstone and carbonate surface more hydrophilic. M. Tariq et al.(Tariq et al., 2019) studied the adsorption and viscoelastic behaviour of ionic liquid surfactants of on gold surfaces. The results showed that the ionic liquids surfactants adsorption quantity was low, and the surfactants aggregation would increase the surfactants adsorption quantity. The surfactants adsorption onto rock surface could be controlled by the surface charge of rocks and surfactants (Liu et al., 2021; Ruan et al., 2021).

Although many researches focus on the behavior of surfactants adsorption on the minerals surface, there are still some shortcomings in the previous study, as was shown as follows: 1) In the previous, most researches were focused on the chemical surfactants adsorption onto carbonate rocks, but the research on biosurfactants adsorption behavior was few. Besides, the cationic surfactant, anionic surfactant and non-ionic surfactant adsorption differences were unclear. 2) Many researches were conducted at ambient conditions, but there exist high temperature and high salinity in actual surfactant enhanced oil recovery process, but the temperature or salinity effect on surfactant adsorption was less. 3) Most researches only focused on the surfactants adsorption behavior, but the methods to decrease surfactants adsorption was few. 4) Molecular dynamics simulation was used to simulate the enhanced oil recovery process (Fan et al., 2018; Koleini et al., 2019b; Dehaghani et al., 2019; Kargozarfard et al., 2020). But the corresponding simulation on surfactants adsorption, surfactants-nanoparticles adsorption was few.

The aim of this research was as follows: 1) to explore surfactants species, temperature, salinity effect on surfactants adsorption onto carbonate rocks surface, and the adsorption isotherms were fitted by Langmuir, Freundlich, Temkin and Linear models; 2) to study SiO_2_ and TiO_2_ nanoparticles effect on decreasing surfactants adsorption; 3) to use molecular dynamics simulation to explore the nanoparticles effect on surfactants adsorption.

## 2 Materials and Methods

### 2.1 Materials

In this study, five surfactants (CTAB, SDS, TX-100, sophorolipid, rhamnolipid) were used in this experiment. Chemical surfactants, CTAB, SDS, TX-100 were from Aladdin, Shanghai. Biosurfactants, sophorolipid and rhamnolipid were from Aladdin, Shanghai. The five surfactants structure was shown in Supplementary Figure S1. The surfactant solution pH was adjusted to 8.0 by the HCl and NaOH. The different inorganic salts (NaCl, CaCl_2_) were purchased from Sigma-Aldrich with 97.0% grade. The carbonate rocks were granule, and the calcium carbonate rocks were crushed, and then was sieved, the particles sizes were in the range of 500–700 μm, and then was washed by deionized water and dried.

### 2.2 Surfactant Solution Preparation

Surfactants could be divided into chemical surfactants and biosurfactants. In addition, surfactants could also be divided into cationic surfactants, anionic surfactants and nonionic surfactants. In order to explore the surfactants adsorption differences between chemical surfactant and biosurfactants, the differences among cationic surfactants, anionic surfactants and non-ionic surfactants, we chose five surfactants CTAB, SDS, TX-100, sophorolipid and rhamnolipid. CTAB, SDS and TX-100 were chemical surfactants, and sophorolipid and rhamnolipid were biosurfactants. Besides, CTAB was cationic surfactant, SDS was anionic surfactant and TX-100 was non-ionic surfactant. The five surfactants included chemical surfactants (CTAB, SDS, TX-100) and biosurfactants (sophorolipid, rhamnolipid). Besides, the CTAB was cationic surfactant, SDS was anionic surfactant, and TX-100 was non-surfactant. We want to explore the adsorption differences between chemical surfactants and biosurfactants. Besides, the cationic surfactant, anionic surfactant and nonionic surfactant adsorption effect differences on the carbonate rocks surface. Therefore, we chose the five surfactants as the representative surfactant.

The different surfactants were used to form the different concentrations of surfactants solution. The surfactants solutions were formed by dissolving the 0.1–5.0 g surfactants into the 1,000 ml deionized water into 1,000 ml-volumetric flask, and then the different concentrations surfactant solutions were formed.

### 2.3 Surfactants Adsorption Experiment

In this study, the surfactants adsorption experiment was conducted to study the surfactants adsorption onto the carbonate rock surface, and the adsorption isotherms and adsorption dynamics were studied (Sun et al., 2011; Alhassawi and Romero-Zeron 2015). The detailed experiment procedures were as follows: 1) Five surfactant solutions were used to prepare the different concentrations solutions. 2) Then 100 ml five surfactants solutions and 10 g carbonate rocks were combined together. 3) The surfactants concentration was measured by the UV-Vis measurement, and the surfactants solution and carbonate rocks were mixed and stirred together, so as to make it mixed uniformly. 4) After the stirring process, the residual surfactants concentration was measured by the UV-Vis measurement.

When the surfactants adsorption quantity remained stable, then the adsorption equilibrium has been reached. In our study, the surfactant adsorption time was within 48 h. Therefore, the surfactants equilibrium concentration and surfactant initial concentration have been explored, the surfactants adsorption quantity could be calculated by the Eq. 1, where q was the surfactant quantity onto the carbonate rock surface (mg/g), m_solution_ (g) was the surfactant mass in the original surfactant solutions, and the c^0^ (mg/L) was the surfactant initial concentration after surfactant adsorption process, c (mg/L) was the surfactant concentration after surfactant adsorption process. In this study, every measurement was repeated at least three times, and the average value was chosen as the data. The experiment was conducted at 25°C.

$$\text{q}=\frac{m_{solution}\left(c^{0}-c\right)}{m_{carbonate}}\times 10^{-3}$$ (1)

### 2.4 Morphology Analysis

After the carbonate rocks were treated by different surfactants, the morphology of the carbonate rocks was observed by SEM-EDS (S4800, Tokyo, Japan) method.

### 2.5 Adsorption Isotherms Models

In order to explore the surfactants adsorption rules, the adsorption models were used, and the four adsorption isotherms were fitted.

#### 2.5.1 Langmuir Model

Langmuir model was used to describe surfactants adsorption behavior onto calcium carbonate surface, which was expressed by as Eq. 2 (Ahmadi and Shadizadeh 2012; Monfared et al., 2015), where q_e_ is the quantity of the surfactant adsorption at the equilibrium per unit mass of calcite (mg/g), Q_m_ is the maximum uptake capacity (mg/g), Ce is the surfactant equilibrium concentration in solution (mg/L), K_L_ is Langmuir constant related to the adsorption site (mg/L).

$${\text{q}}_{e}=\frac{Q_{m}K_{L}C_{e}}{1+K_{L}C_{e}}$$ (2)

The Langmuir model assumptions were as follows (Barati et al., 2016): The calcite surface should be considered homogeneous. Besides, surfactants molecules should have equal molar surface areas. Bulk and surface phase show an ideal behavior.

#### 2.5.2 Freundlich Model

Freundlich model was based on the assumption that the calcium carbonate surface had a heterogeneous surface and different classes of adsorption sites covered the surface (Arabloo et al., 2015; Barati et al., 2016).

Freundlich model is expressed by Eq. 3 (Bera et al., 2013), where q_e_ is the quantity of the surfactants adsorption at the equilibrium per unit mass of calcium carbonate surface (mg/g), Ce is the surfactant equilibrium concentration in solution (mg/L), K_F_ Freundlich constants (L/mg), which showed the surfactants adsorption capacity, n is related to the adsorption intensity.

$${\text{q}}_{e}=K_{F}C_{e}^{\frac{1}{n}}$$ (3)

#### 2.5.3 Temkin Model

The assumption of the Temkin model was that during the adsorption process the heat of adsorption decreases linearly and is not a function of logarithmic (Saxena et al., 2018). The Temkin model is expressed as the Eq. 4 (Ahmadi and Shadizadeh 2015; Saxena et al., 2018), where q_e_ is the quantity of the surfactant adsorption at the equilibrium per unit mass of calcium carbonate (mg/g), Ce is the surfactant equilibrium concentration in solution (mg/L), B is the Temkin constant, K_T_ is the equilibrium binding (L/mg), respectively.

$${\text{q}}_{e}=B\ln\left(K_{T}C_{e}\right)$$ (4)

#### 2.5.4 Linear Model

The Linear model was the most simplified model, which was expressed as Eq. 5 (Barati et al., 2016), where q_e_ is the quantity of the surfactant adsorption at the equilibrium per unit mass of calcium carbonate (mg/g), K_H_ was the linear constant (L/mg), Ce is the surfactant equilibrium concentration in solution (mg/L),

$${\text{q}}_{e}=K_{H}C_{e}$$ (5)

### 2.6 Contact Angle Measurement

The contact angle was measured to value the carbonate reservoirs wettability alteration by different surfactants adsorption. Calcite surface was used to represent the carbonate rocks. Five surfactants with different species, concentration, temperature and salinity were used to treat the calcite surface for 3 days. Then the water drop was dripped onto the calcite surface, and the initial contact angle was the static contact angle. When calcite surface was immersed into the aqueous phase, the oil drop was injected into the calcite surface, and then the contact angle alteration with time was measured, which was the dynamic contact angle.

### 2.7 Surface Tension Measurement

In this study, the five surfactant solutions (2000 ppm) and five surfactants (2000 ppm)-SiO_2_ nanoparticles (0.5 wt%) solutions were measured by the programmable tensiometer (Kruss GmbH, Germany, Model: K20 EasyDyne) at 298 K by the Du Noüy ring method. Every experiment was repeated three times, and the average value was the surface tension.

## 3 Simulation Section

In this study, the molecular dynamics simulation was conducted to value the five surfactants (CTAB, SDS, TX-100, Sophorolipid, Rhamnolipid) adsorption behavior onto the carbonate rocks surface. In recent years, calcite was used to represent the carbonate rocks during the molecular dynamics simulation process (Badizad et al., 2020a). The simulation software was Materials Studio 8.0. The COMPASS force field was applied during the simulation process. Based on the COMPASS force field, the total energy was shown in Eq. 6 (Yuan et al., 2016). The total energy (*E*_*total*_) could be divided into two parts: valence terms and nonbond interaction terms (Sun et al., 1998). The valence terms included bond energy ( $\sum_{bond}E_{b}\left(b\right)$ ), angle energy ( $\sum_{angle}E_{\theta }\left(\theta \right)$ ), torsion energy ( $\sum_{out-of-plane}E_{\chi }\left(\chi \right)$ ), out-of-plane energy ( $\sum_{out-of-plane}E_{\chi }\left(\chi \right)$ ), and cross-coupling energy ( $\sum_{cross}E\left(b,\theta ,\varphi \right)$ ). The nonbond interaction terms included van der Waals energy ( $E_{vdw}$ ) Eq. 8 and Columbic interaction ( $E_{coulomb}$ ) (Eq. 7, where q_i_ and q_j_ are the charges of atoms i and j, with a distance r_ij_, and e is the potential well depth for the interaction between the two atoms.

Etotal=∑bondEb(b)+∑angleEθ(θ)+∑dihedralEφ(φ)+∑out−of−planeEχ(χ)+∑crossE(b,θ,φ)+E+coulombEvdw (6)

$$E_{coulomb}=\sum_{i>j}\frac{q_{i}q_{j}}{r_{ij}}$$ (7)

$$E_{vdw}=\sum \varepsilon_{ij}\left[2{\left(\frac{r_{ij}^{0}}{r_{ij}}\right)}^{9}-3{\left(\frac{r_{ij}^{0}}{r_{ij}}\right)}^{6}\right]$$ (8)

The detailed surfactants adsorption behavior was as follows:

1) Cleave the calcite (1 0 4) surface as the calcium carbonate surface. Then the calcite (1 0 4) surface was conducted the energy optimization and structure optimization, so as to obtain the stablest conformation, and fixed the calcite surface (1 0 4). The Ca atom and C atom were spatially constrained to avoid distortion or deformation of the slits walls, which was because of CaCO_3_ thermodynamic and kinetic characteristics basis (Badizad et al., 2020b).
2) Build the corresponding supercell, and the vacuum layer thickness was 50 Å. Besides, the periodicity changed from the two-dimensional to three-dimensional. The corresponding cell parameters were *a* = 72.86228 Å, *b* = 29.940008 Å, and the corresponding interfacial angle was *α* = 90°, *β* = 90°, *γ* = 90°.
3) Put twenty surfactant molecules into the amorphous cell, then run the COMPASS field of force. The COMPASS forcefield was used in the whole simulation section, and the surfactants adsorption process, surfactants-nanoparticles adsorption was conducted using COMPASS forcefield (Koleini et al., 2020).
4) Build layers, layer 1 was calcite surface, layer 2 was surfactants unit cell, and surfactants were used to adsorb onto the calcite surface, and the adsorption time continued 2000 ps, the step was 1fs. The simulation process conditions were as follows: run module was Forcite, NVT ensemble, COMPASS force field, cutoff distance 12.5 Å, 298 K, Berendsen thermostat.
5) Then the SiO_2_ nanoparticles were added into the simulation, and the simulation procedure followed the above process. Then the surfactants-nanoparticles system was conducted. The diffusion coefficients and concentration profile of surfactants molecules were analyzed. The diffusion coefficients of surfactants molecules onto the calcite surface are calculated by Eq. 9, where MSD represents the mean-square displacement (Å^2^), N is surfactants molecules number, R_i_(t) is the coordinate of atom i at time of t, and R_i_ (0) is the initial position of atom.

$$MSD=\sum_{i}^{N}<{\left[R_{i}\left(t\right)-R_{i}\left(0\right)\right]}^{2}>$$ (9)

6) The interaction energy between surfactants and calcite surface at different simulation time was calculated by Eq. 10, where *E*_*interaction energy(surfactant/calcite)*_ is the interaction energy between the surfactant molecules and calcite surface, E_total_ is the energy of the system, including the surfactants and the calcite surface, E_surfactant_ is the energy of the surfactant molecules without calcite surface, E_calcite_ is the energy of the calcite surface without oil molecules, respectively. The interaction energy between surfactants and nanoparticles at different simulation time was calculated by Eq. 11, where *E*_*interaction energy(surfactant/nanoparticles)*_ is the interaction energy between the surfactant molecules and nanoparticles, E_total_ is the energy of the system, including the surfactants and the nanoparticles, E_surfactant_ is the energy of the surfactant molecules without nanoparticles, E_nanoparticles_ is the energy of the calcite surface without nanoparticles, respectively.

$$E_{\begin{array}{l} \mathrm{interaction}\;\mathrm{energy}\left(\mathrm{surfactant/calcite}\right) \end{array}}=E_{total}-\left(E_{surfac\tan t}+E_{calcite}\right)$$ (10)

$$E_{\begin{array}{l} \mathrm{interaction}\;\mathrm{energy}\left(\mathrm{surfactant/nanoparticles}\right) \end{array}}=E_{total}-\left(E_{surfac\tan t}+E_{nanoparticles}\right)$$ (11)

## 4 Results and Discussion

### 4.1 Surfactants Adsorption at Ambient Conditions

**FIGURE 1:**
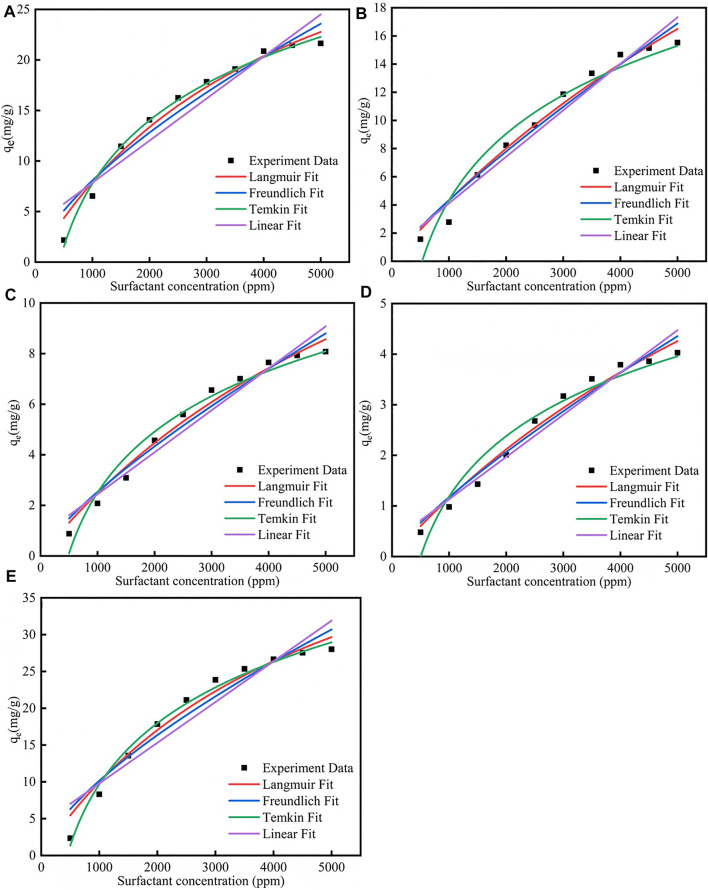
Different isotherm models fit for adsorption of **(A)** CTAB, **(B)** SDS, **(C)** TX-100, **(D)** sophorolipid, **(E)** rhamnolipid on carbonate rock at 298 K.

Figure 1; Supplementary Table S1 showed the different isotherm models fit for adsorption of five surfactants onto carbonate rock at 298 K. The Q_m_ parameter of the CTAB, SDS, TX-100, sophorolipid and rhamnolipid were 28.12 mg/g, 24.81 mg/g, 13.45 mg/g, 7.18 mg/g, 43.57 mg/g, respectively, which means that the surfactants adsorption quantity onto the calcite surface followed the rule: rhamnolipid > CTAB > SDS > TX-100 > sophorolipid. Besides, the *R* ^2^ of the five surfactants for Langmuir, Freundlich and Temkin was well, but the *R* ^2^ for the Linear fit was bad, which means that the Linear model was not fitted the surfactant adsorption. The B for Temkin model by the five surfactants were 11.9857 (rhamnolipid) >9.015(CTAB)> 6.818(SDS)> 6.818 (TX-100)> 1.7207 (sophorolipid), which was in accordance with the surfactants adsorption quantity. In other words, when the B value was higher, the surfactants adsorption quantity would increase.

### 4.2 SEM Analysis

The calcite surfaces after surfactants adsorption were shown in Supplementary Figure S2. As was shown in Supplementary Figure S2A, when CTAB adsorbed onto the calcite surface, the surface was flat, and there was not the obvious shape alteration. Although CTAB adsorption was big, but the calcite shape alteration was not obvious. When SDS was used to alter the calcite surface, the surface become rugged. Other surfactants showed the similar effect like SDS.

### 4.3 Temperature Effect on Surfactants Adsorption

**FIGURE 2:**
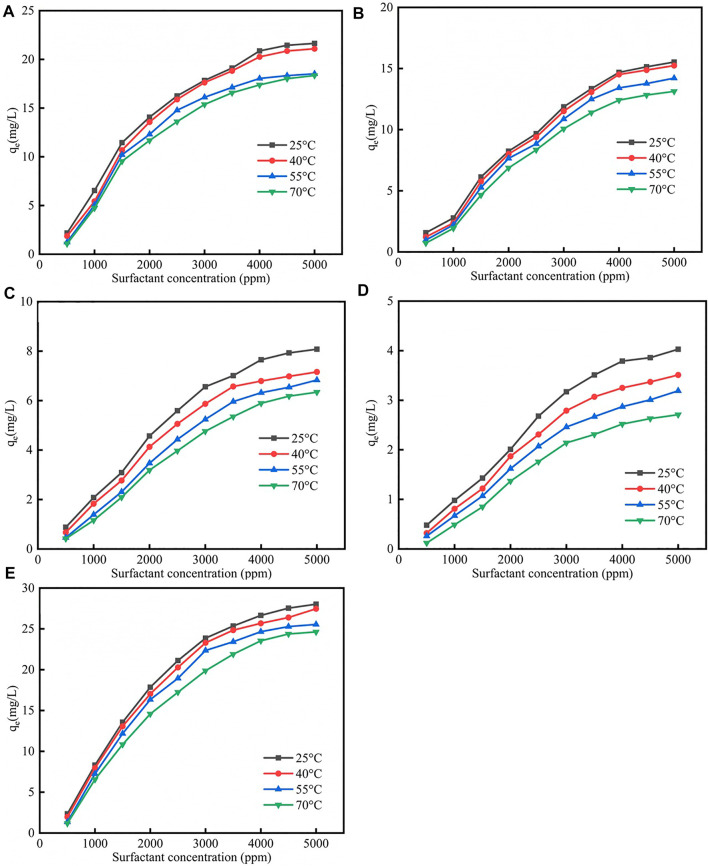
Different surfactants **(A)** CTAB, **(B)** SDS, **(C)** TX-100, **(D)** sophorolipid, **(E)** rhamnolipid adsorption on carbonate rock at different temperatures.

Due to the fact that the enhanced oil recovery was conducted at reservoir environment, therefore the temperature effect on surfactants adsorption was studied (Badizad et al., 2020). Figure 2 showed the different isotherm models fit for adsorption of five surfactants on calcite at different temperatures were shown in Supplementary Figures S3–S7; Supplementary Tables S2–S6. As was shown in Figure 2, the surfactants adsorption quantity would decrease when the temperature increased, the reason was when the temperature increased, the molecular thermal motion rate would increase, which helped the surfactant desorption. As was shown in Supplementary Tables S2–S6, when the temperature increased the Q_m_ would decrease, and the corresponding rate would decrease.

### 4.4 Salinity Effect on Surfactants Adsorption

**FIGURE 3:**
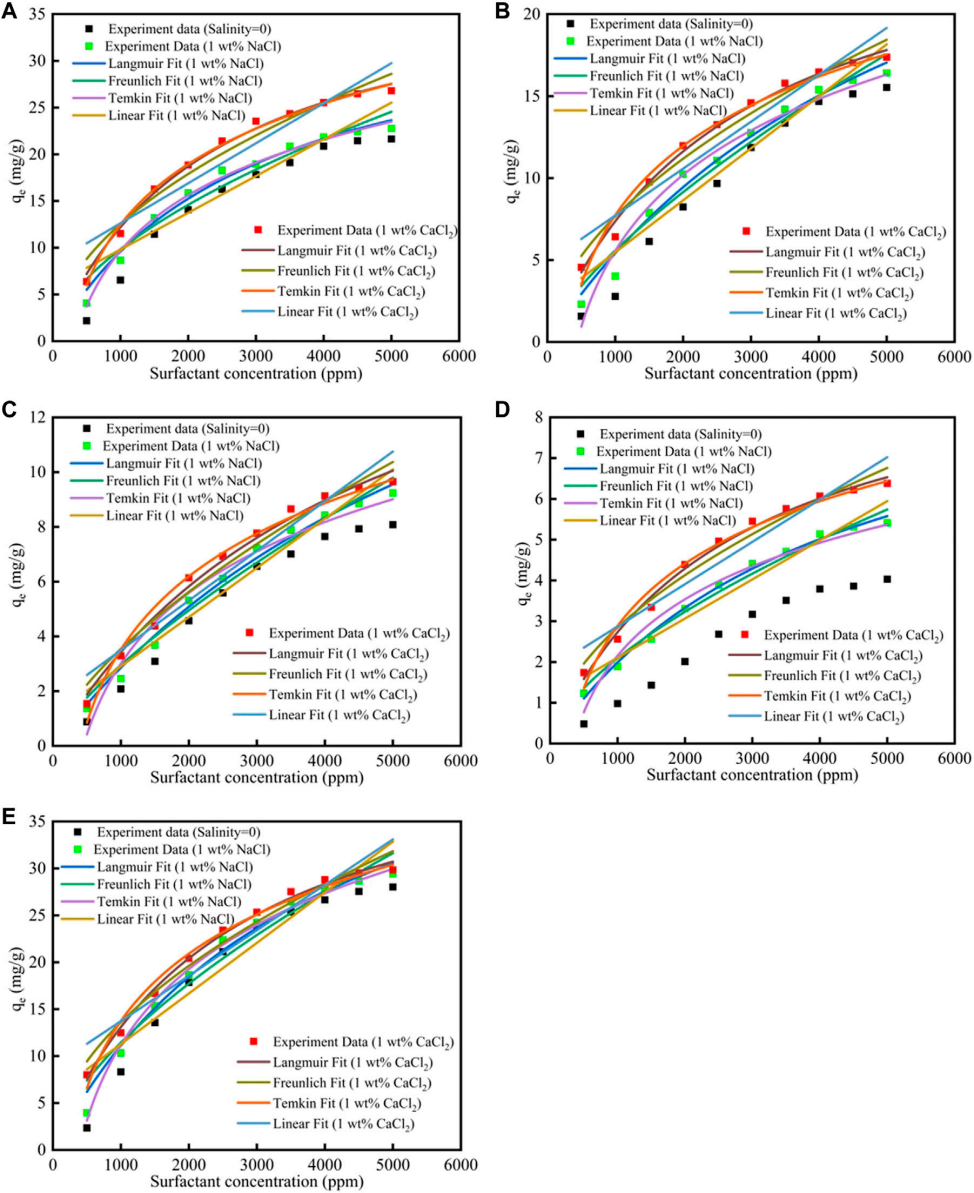
Different surfactants **(A)** CTAB, **(B)** SDS, **(C)**TX-100, **(D)** sophorolipid, **(E)** rhamnolipid adsorption on carbonate rock at different salinities.

The salinity effect on surfactants adsorption was shown in Figure 3; Supplementary Tables S7, S8. When the salinity increased, the surfactants adsorption quantity would increase, and the reason was because that the salinity would compress the electric double layer, which helped the surfactants adsorption. The *R* ^2^ of the Langmuir fit for the five surfactants were good, which means that the Langmuir fitness was good, and the surfactants adsorption were more suitable for the Langmuir model.

The ionic strength could be calculated by Eq. 12, where I represent ionic strength (mol/kg), c_i_ represent the i ion concentration (mol/kg), and z_i_ was the ion charge. Debye-Hückel theory calculated the activity quotient, shown in Eq. 13, where $\gamma_{\pm }$ was the activity quotient, I was the ionic strength, and z_+_ and z_−_ were the anion ions and cationic ions charge, A was 0.509 mol^0.5^kg^0.5^ (25°C). For 1 wt% NaCl solution, the ionic strength was 0.171 mol/kg, and the $\gamma_{\pm }$ was 0.616 mol/kg. For 1 wt% CaCl_2_ solution, the ionic strength was 0.270 mol/kg, and the $\gamma_{\pm }$ was 0.296 mol/kg. The Ca^2+^ showed higher ionic strength, and could compress the electric double layer higher than Na^+^, and then the calcite surface charge change was higher than the Na^+^(Jian et al., 2018). The surfactants adsorption quantity would increase higher in divalent ions solutions.

$$I=\frac{1}{2}\sum_{i=1}^{n}c_{i}z_{i}^{2}$$ (12)

$$\mathrm{lg}\gamma_{\pm }=-A/{\text{z}}_{+}z_{-}/\sqrt{I}$$ (13)

### 4.5 Nanoparticles Effect on Surfactants Adsorption

**FIGURE 4:**
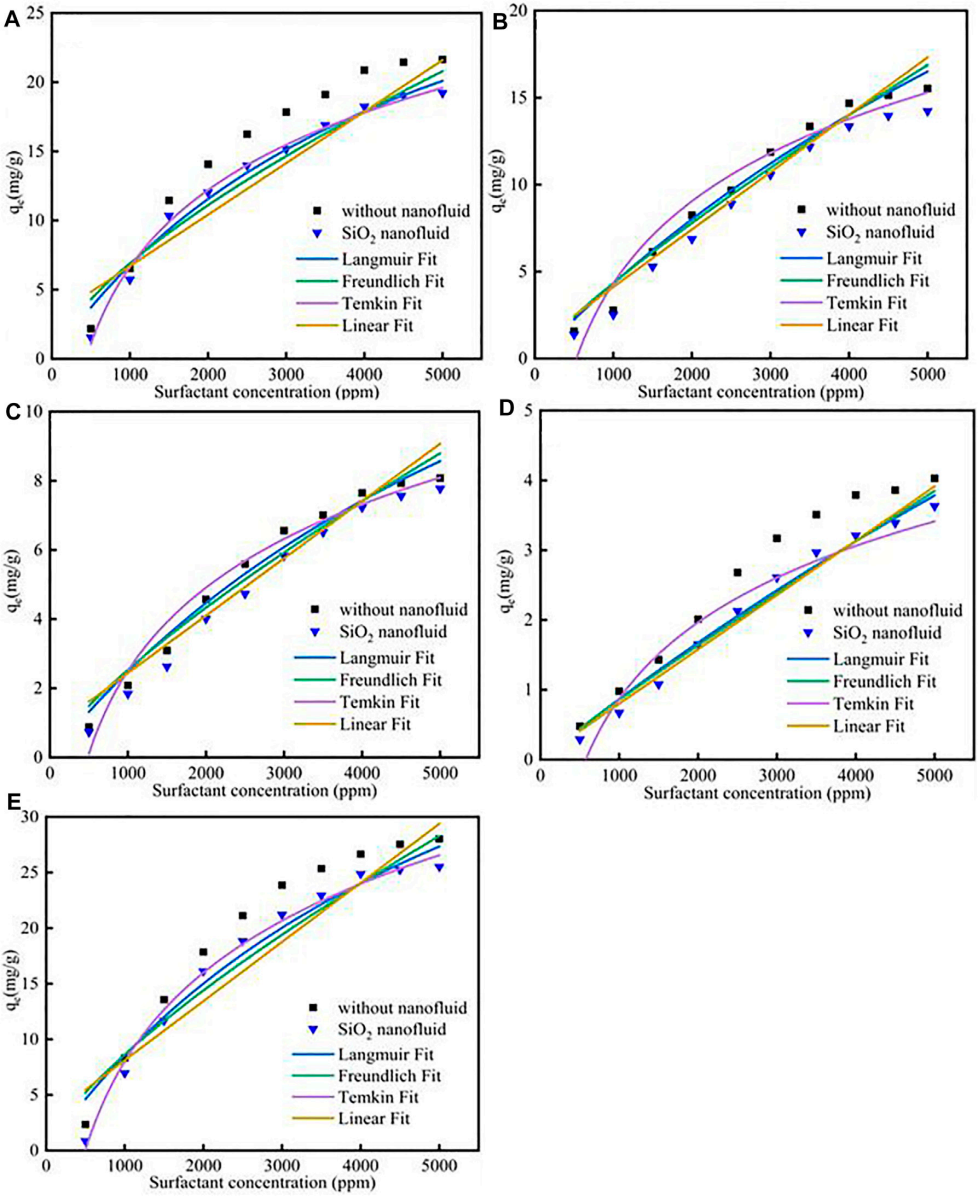
Different surfactants **(A)** CTAB, **(B)** SDS, **(C)**TX-100, **(D)** sophorolipid, **(E)** rhamnolipid with SiO_2_ nanoparticles adsorption on carbonate rock at 298 K.

Different fitness of surfactants with SiO_2_ nanoparticles adsorption on calcite at 298 K was shown in Figure 4; Supplementary Table S9. The results showed that the SiO_2_ nanoparticles could effectively decrease the surfactants adsorption. The reason was because surfactants could effectively adsorb onto SiO_2_ nanoparticles surface, and then the surfactants adsorption would decrease, the adsorption effect could be verified by the molecular dynamic simulation. For Langmuir model, the Q_m_ for CTAB, SDS, TX-100, sophorolipid and rhamnolipid were 39.57 mg/g, 38.16 mg/g, 21.87 mg/g, 9.72 mg/g, 46.18 mg/g, respectively. The adsorption quantity follows the previous surfactants adsorption procedure. The K_L_ for the five surfactants were 2.06 × 10^−4^, 6.27 × 10^−5^, 7.31 × 10^−5^, 3.86 × 10^−5^, 1.66 × 10^−4^, respectively. For Freundlich model, the K_F_ value was 6.12 × 10^−2^, 8.52 × 10^−3^, 5.7 × 10^−3^, 1.36 × 10^−3^, 5.33 × 10^−2^, respectively. The value was lower than the corresponding value in high salinity, therefore, it shows that for the Freundlich model, the surfactants adsorption was still less.

**FIGURE 5:**
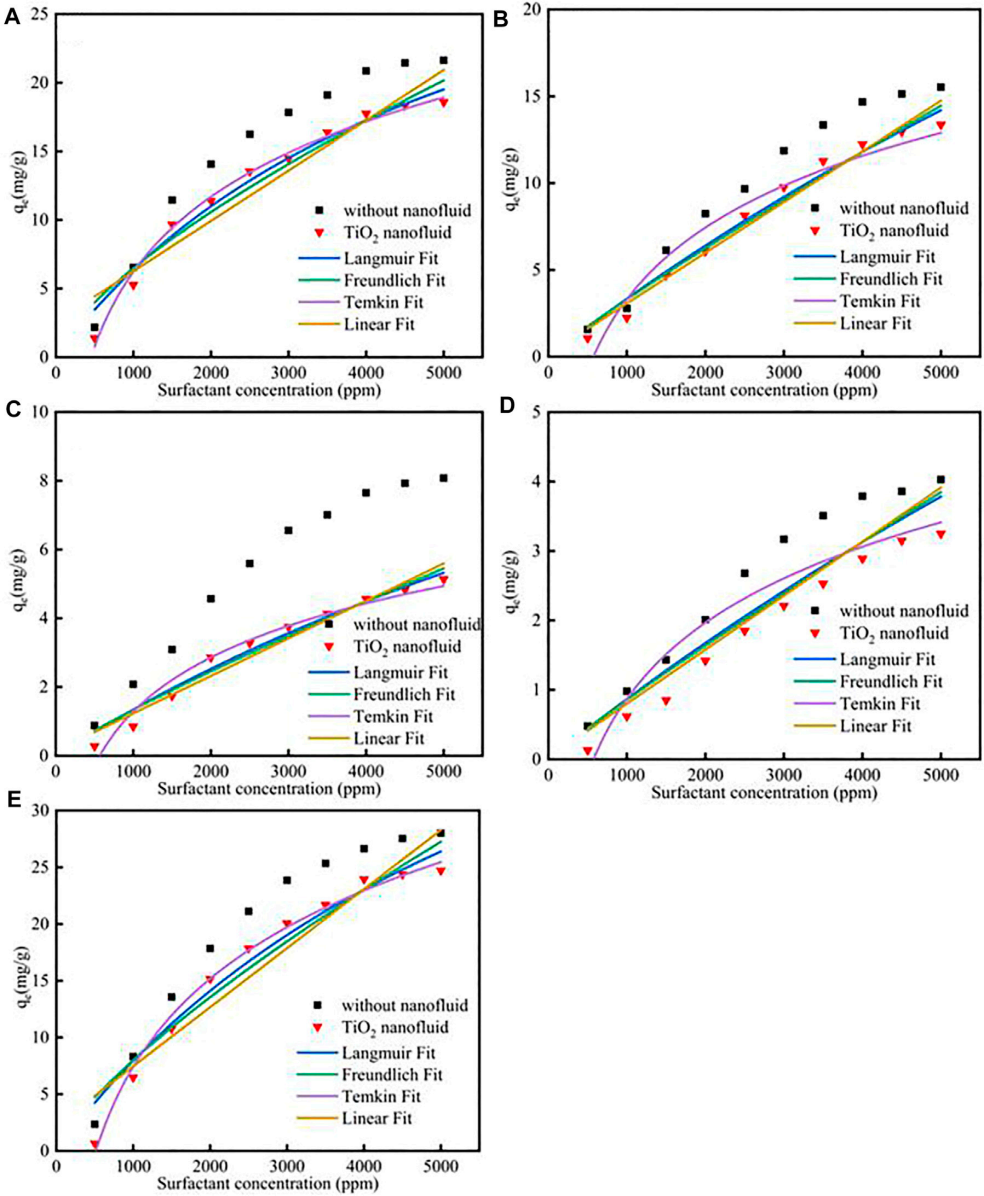
Different surfactants **(A)** CTAB, **(B)** SDS, **(C)**TX-100, **(D)** sophorolipid, **(E)** rhamnolipid with TiO_2_ nanoparticles adsorption on carbonate rock at 298 K.

Different fitness of surfactants with TiO_2_ nanoparticles adsorption on calcite at 298 K was shown in Figure 5; Supplementary Table S10. The results showed that the TiO_2_ nanoparticles could also decrease the surfactants adsorption. Besides, the correspond values of the different surfactant adsorption K_L_, K_F_, K_T_, K_H_ would decrease.

### 4.6 Molecular Dynamics Analysis

#### 4.6.1 Adsorption Snapshot Analysis

**FIGURE 6:**
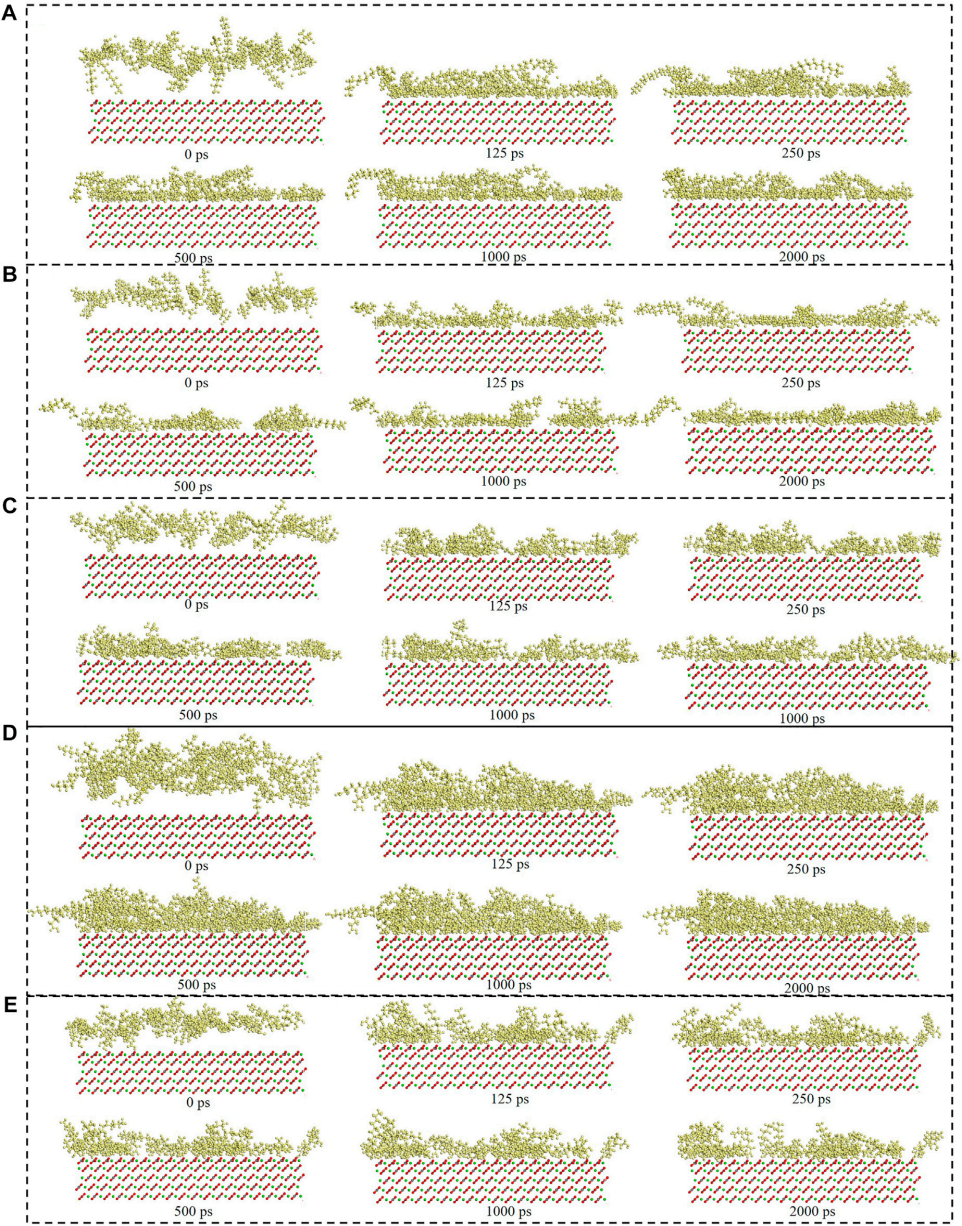
Consecutive snapshot (0 ps–2000 ps) of **(A)** CTAB, **(B)** SDS, **(C)** TX-100, **(D)** Sophorolipid, **(E)** Rhamnolipid adsorption onto the modelled calcite surface. yellow = surfactants, red and grey = CO_3_^2-^, green = Ca^2+^, red, grey and green = calcite surface.

**FIGURE 7:**
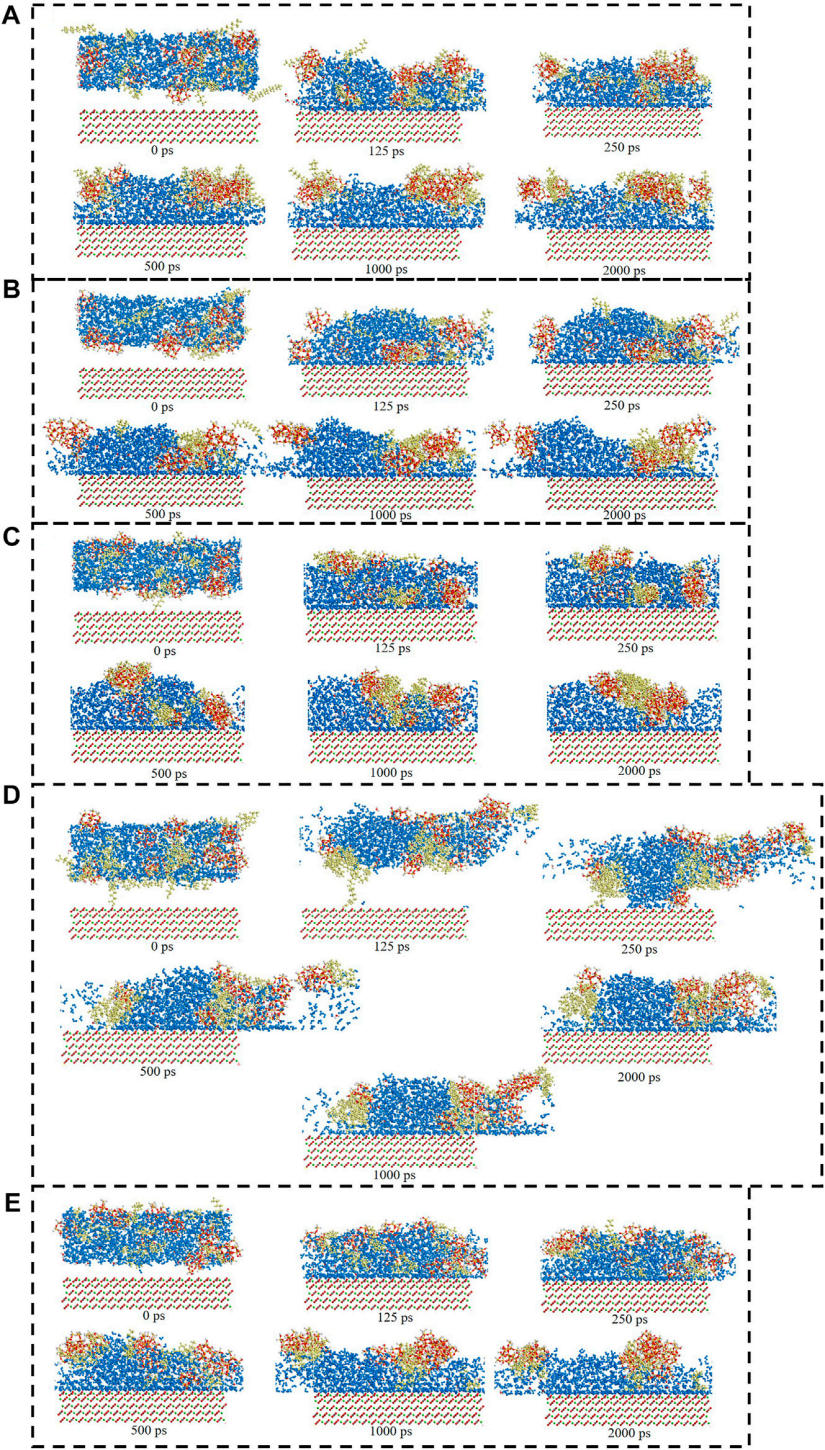
Consecutive snapshot (0 ps–2000 ps) of **(A)** CTAB-SiO_2_, **(B)** SDS-SiO_2_, **(C)** TX-100-SiO_2_, **(D)** Sophorolipid-SiO_2_, **(E)** Rhamnolipid-SiO_2_ adsorption onto the modelled calcite surface. yellow = surfactants, red and grey = CO_3_^2−^, green = Ca^2+^, red, grey and green = calcite surface, red and white = SiO_2_ nanoparticles.

Figure 6 showed the surfactants adsorption snapshot at different internals (0ps-2000 ps). As was shown in this Figure 6, the five surfactants adsorption behavior was similar. When the simulation time was 125 ps, the five surfactants could adsorb onto the calcite surface, and when the simulation time continues, the surfactants adsorption conformation would be altered. In the end, all the five surfactants could remain the stable adsorption behavior. Figure 7 showed the different surfactants-SiO_2_ nanoparticles adsorption snapshot at different internals. The experiment results showed that the nanoparticles could efficiently decrease surfactants adsorption effect, and in order to explain the corresponding mechanism, the water phase was added in the simulation process (Koleini et al., 2021). As was shown in Figure 7A to Figure 7E, the surfactants would adsorb onto SiO_2_ nanoparticles surface, which would make the surfactants adsorption quantity would decrease. When the time was 125 ps, some surfactants molecules would contact with the SiO_2_ nanoparticles, and when the simulation time proceeds, the surfactants molecules would closely with the SiO_2_ nanoparticles. The simulation results (Figure 7) showed that five surfactants could adsorb onto nanoparticles surface, increase the steric hindrance between different surfactants, and then decrease surfactants adsorption onto calcium carbonate surface. The results were in accordance with the experiment results.

The surfactants adsorption behavior included two parts: one the one hand, the intermolecular force, which was verified by interaction energy data. One the other hand, the electrostatic force influenced the molecules behavior by coulomb action. Besides, salinity would influence the surfactant-calcite carbonate interaction force. The heavy oil-calcium carbonate interfacial behavior was influenced by the CaCO_3_ mineral surface chemistry and polar hydrocarbons residing charged (Badizad et al., 2021). The surfactant adsorption onto the calcite surface was also influenced by the CaCO_3_ mineral surface chemistry and polar hydrocarbons residing charge.

#### 4.6.2 Surfactants Concentration Analysis and Diffusion Coefficient Analysis

**FIGURE 8:**
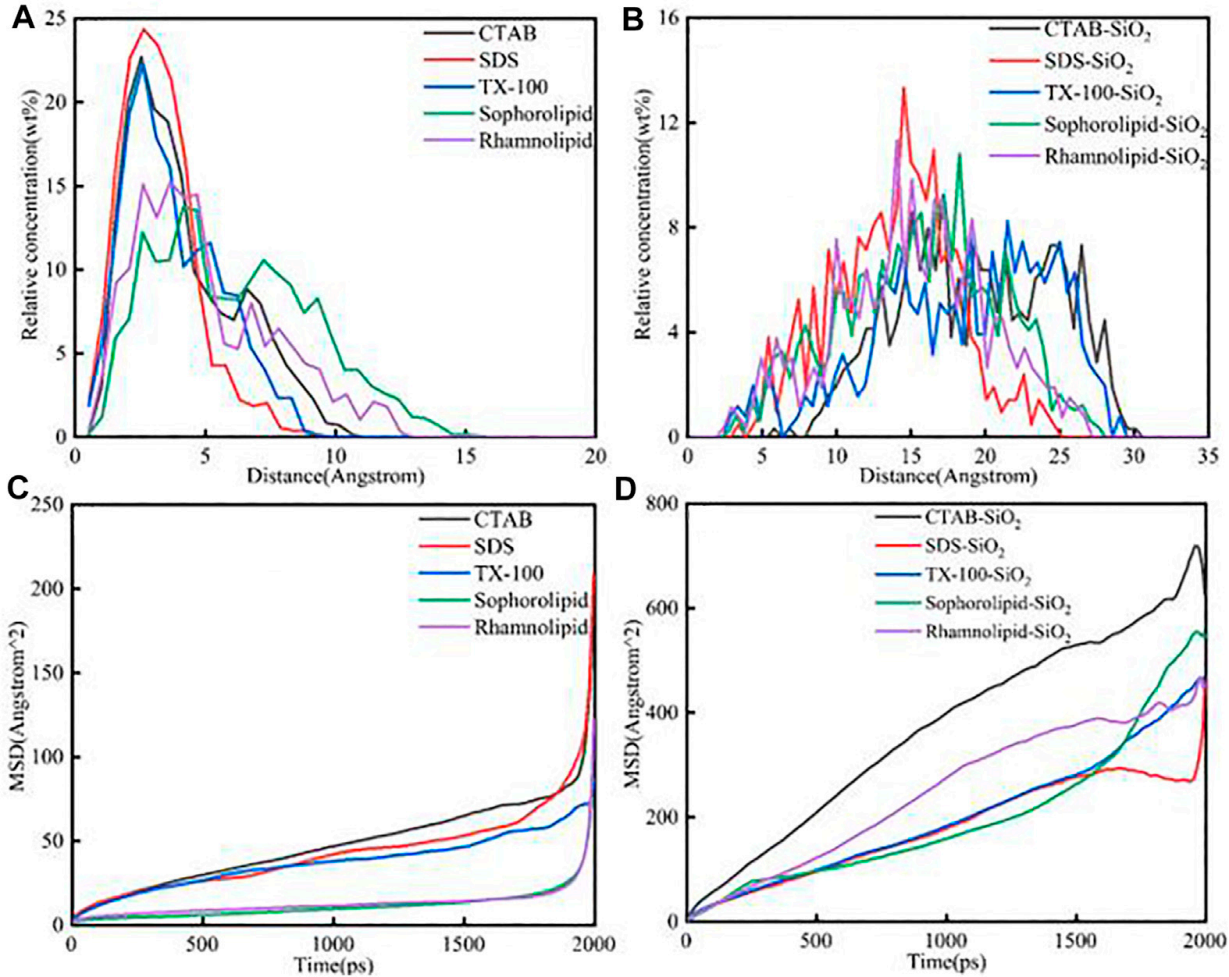
The relative concentration of different **(A)** surfactants; **(B)** surfactants-SiO_2_ nanofluids system. The mean square displacement of different **(C)** surfactants; **(D)** surfactants-SiO_2_ nanofluids system.

The relative concentration and mean square displacement of different surfactants and surfactants-SiO_2_ nanofluids system was shown in Figure 8. Figure 8A showed that the peak position concentrations of the five surfactants were as follows: TX-100 < SDS < CTAB < rhamonilipid < sophorolipid. As was shown in Figure 8 (c), the mean square displacement order of five surfactants was as follows: CTAB > SDS > TX-100 > rhamnolipid≈sophorolipid (t<1900 ps). When the simulation time was higher than 1900 ps, the mean square displacement was CTAB < SDS. Figure 8 showed that the chemical surfactants showed obvious strong adsorption with the calcite surface. For the surfactants-SiO_2_ nanoparticles system, the distance was longer than the surfactants-calcite surface directly, which means that the surfactants were not closely to the calcite surface. As was shown in Figure 8B, the CTAB and TX-100 was the most far away to the calcite surface. As was shown in Figure 8D, the mean square displacement of CTAB was much higher than other surfactants, which means the CTAB showed obvious effect on decrease the surfactants adsorption.

#### 4.6.3 Energy/Temperature Balance

**FIGURE 9:**
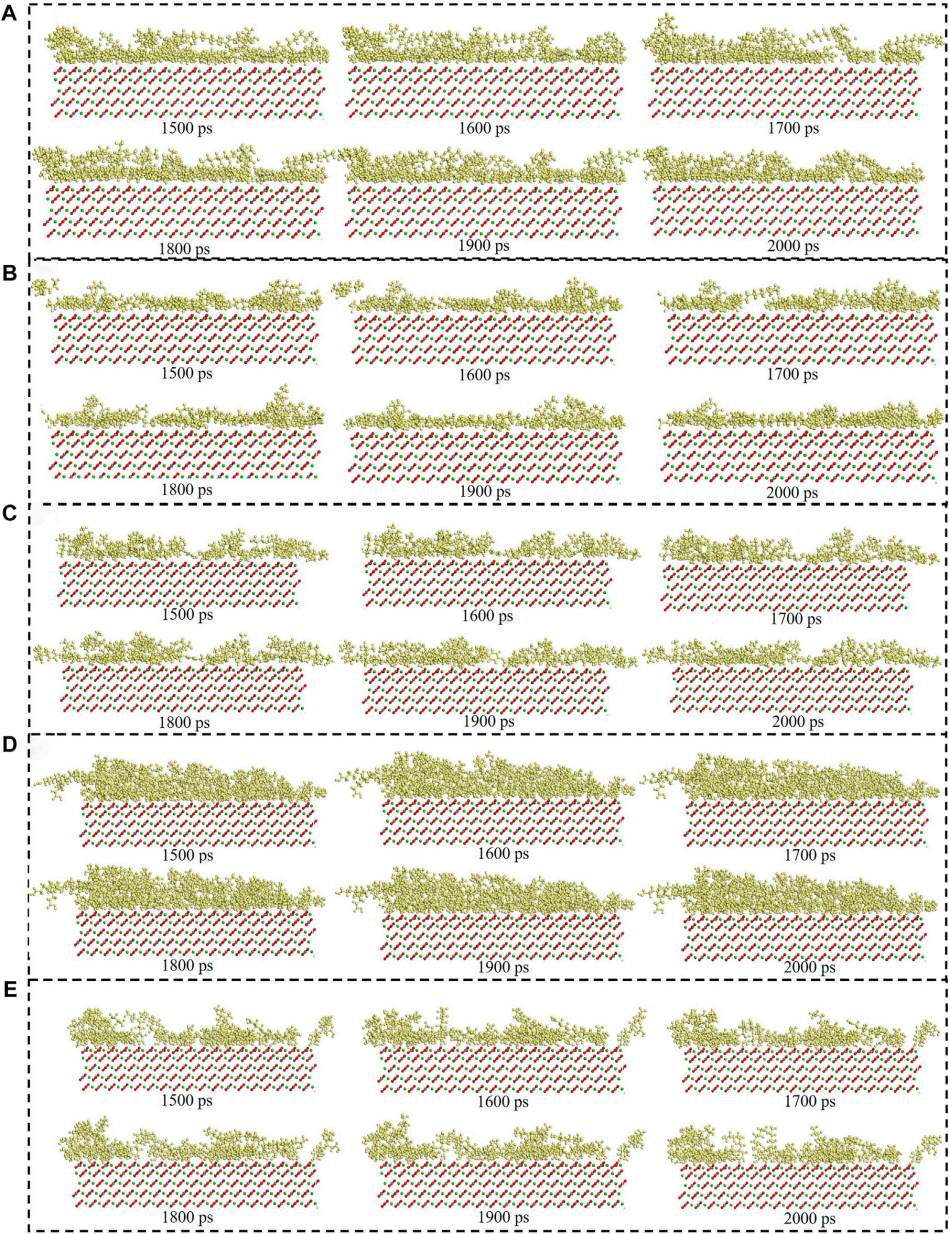
Consecutive snapshot (1,500–2000 ps) of **(A)** CTAB, **(B)** SDS, **(C)** TX-100, **(D)** Sophorolipid, **(E)** Rhamnolipid adsorption onto the modelled calcite surface. yellow = surfactants, red and grey = CO_3_^2-^, green = Ca^2+^, red, grey and green = calcite surface.

**FIGURE 10:**
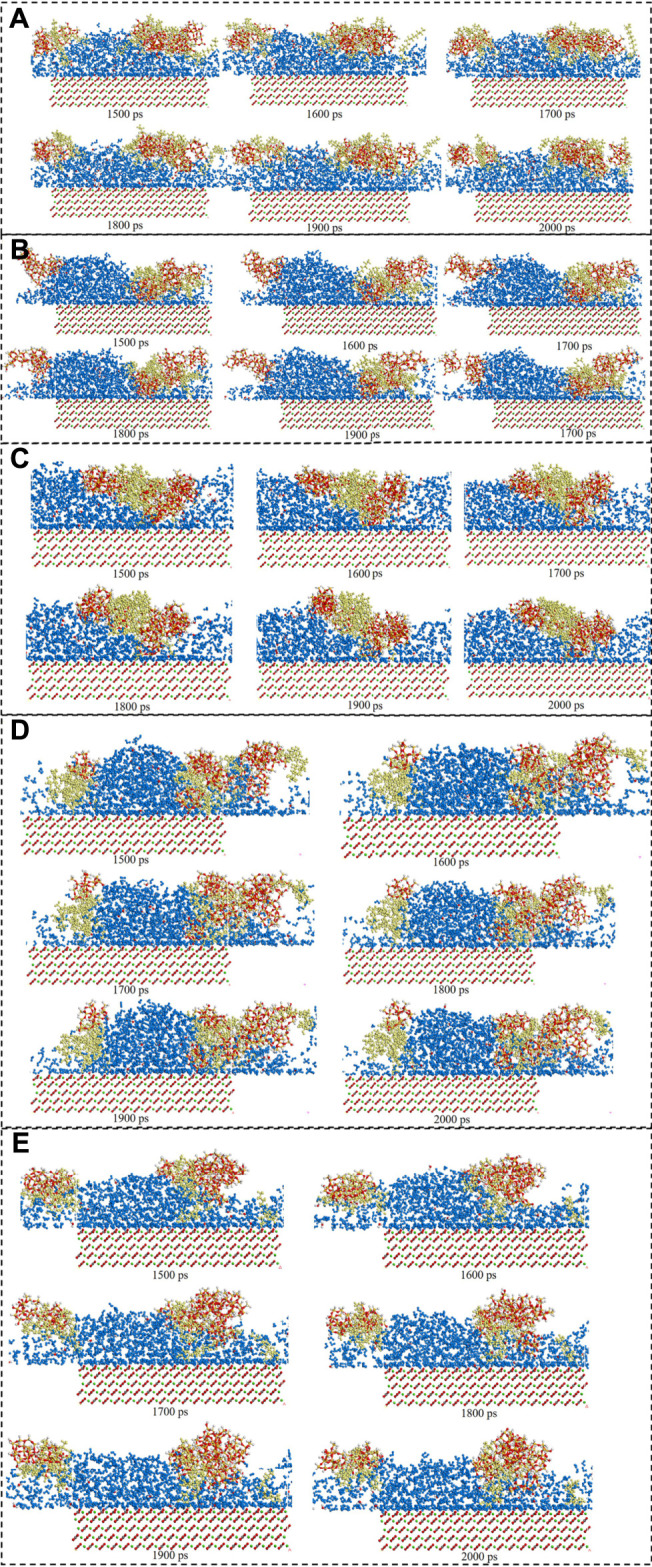
Consecutive snapshot (1,500–2000 ps) of **(A)** CTAB-SiO_2_, **(B)** SDS-SiO_2_, **(C)** TX-100-SiO_2_, **(D)** Sophorolipid-SiO_2_, **(E)** Rhamnolipid-SiO_2_ adsorption onto the modelled calcite surface. yellow = surfactants, red and grey = CO_3_^2−^, green = Ca^2+^, red, grey and green = calcite surface, red and white = SiO_2_ nanoparticles.

The consecutive snapshots (1,500 ps–2000 ps) of five surfactants adsorption onto the modelled calcite surface were shown in Figure 9. The consecutive snapshots (1,500–2000 ps) of five surfactants-SiO_2_ nanoparticles adsorption onto the modelled calcite surface were shown in Figure 10. Figures 9, 10 showed that both the surfactants and surfactants-SiO_2_ nanoparticles adsorption was stable after 1,500 ps, in other words, the position of the surfactant molecules and SiO_2_ nanoparticles remained stable after 1,500 ps

Supplementary Figures S8, S10 showed the energy balance of surfactants-calcite system and surfactants-SiO_2_ nanoparticles calcite system. As was shown in Supplementary Figures S8, S10, the red line represents kinetic energy, and the kinetic energy remained stable, and non-bond energy, potential energy and total energy decreased with simulation time proceeded. The non-bond energy, potential energy, and total energy decreased significantly within 100 ps, and then three energies would become stable. Supplementary Figures S9, S11 showed that the temperature stabilized at 298K, and the temperature fluctuation range was within 10 K. Energy change rate from 1,500 ps to 2000ps could be calculated by Eq. 14, and temperature change rate from 1,500 ps to 2000 ps could be calculated by Eq. 15. Table 1 showed the energy change rate and temperature change rate (%) (1,500 ps–2000 ps) of surfactants (with and without SiO_2_ nanoparticles) system. The temperature change rate for the five surfactants (with and without SiO_2_ nanoparticles) was low. The potential energy, kinetic energy, non-bond energy and total energy change rate were also low.

$$\mathrm{Energy}\;\mathrm{change}\;\mathrm{rate}\left(\text{\%}\right)=\frac{/Energy_{2000ps}-Energy_{1500ps}/}{Energy_{1500ps}}\times 100\%$$ (14)

$$\mathrm{Temperature}\;\mathrm{change}\;\mathrm{rate}\left(\text{\%}\right)=\frac{/Temperature_{2000ps}-Temperature_{1500ps}/}{Temperature_{1500ps}}\times 100\%$$ (15)

**TABLE 1:** The energy change rate and temperature change rate (%) (1,500–2000 ps) of surfactants (with and without SiO_2_ nanoparticles) system.

Surfactants		Potential energy	Kinetic energy	Non-bond energy	Total energy	Temperature
CTAB	Without SiO_2_	3.46	2.98	1.67	1.06	2.97
	With SiO_2_	0.48	1.05	0.04	0.38	1.07
SDS	Without SiO_2_	0.59	3.19	0.06	0.18	3.17
	With SiO_2_	0.22	0.31	0.01	0.21	0.30
TX-100	Without SiO_2_	5.66	2.75	9.78	5.91	1.75
	With SiO_2_	0.56	0.24	0.86	0.70	0.23
Sophoroli-pid	Without SiO_2_	6.29	3.00	19.42	2.87	2.97
	With SiO_2_	0.40	1.61	0.28	0.15	1.61
Rhamnoli-pid	Without SiO_2_	1.59	9.80	1.12	58.2	3.79
	With SiO_2_	0.18	2.25	0.47	0.61	2.25

## 5 The Comparison Analysis Between Simulation and Experiments

### 5.1 Contact Angle Measurement

**FIGURE 11:**
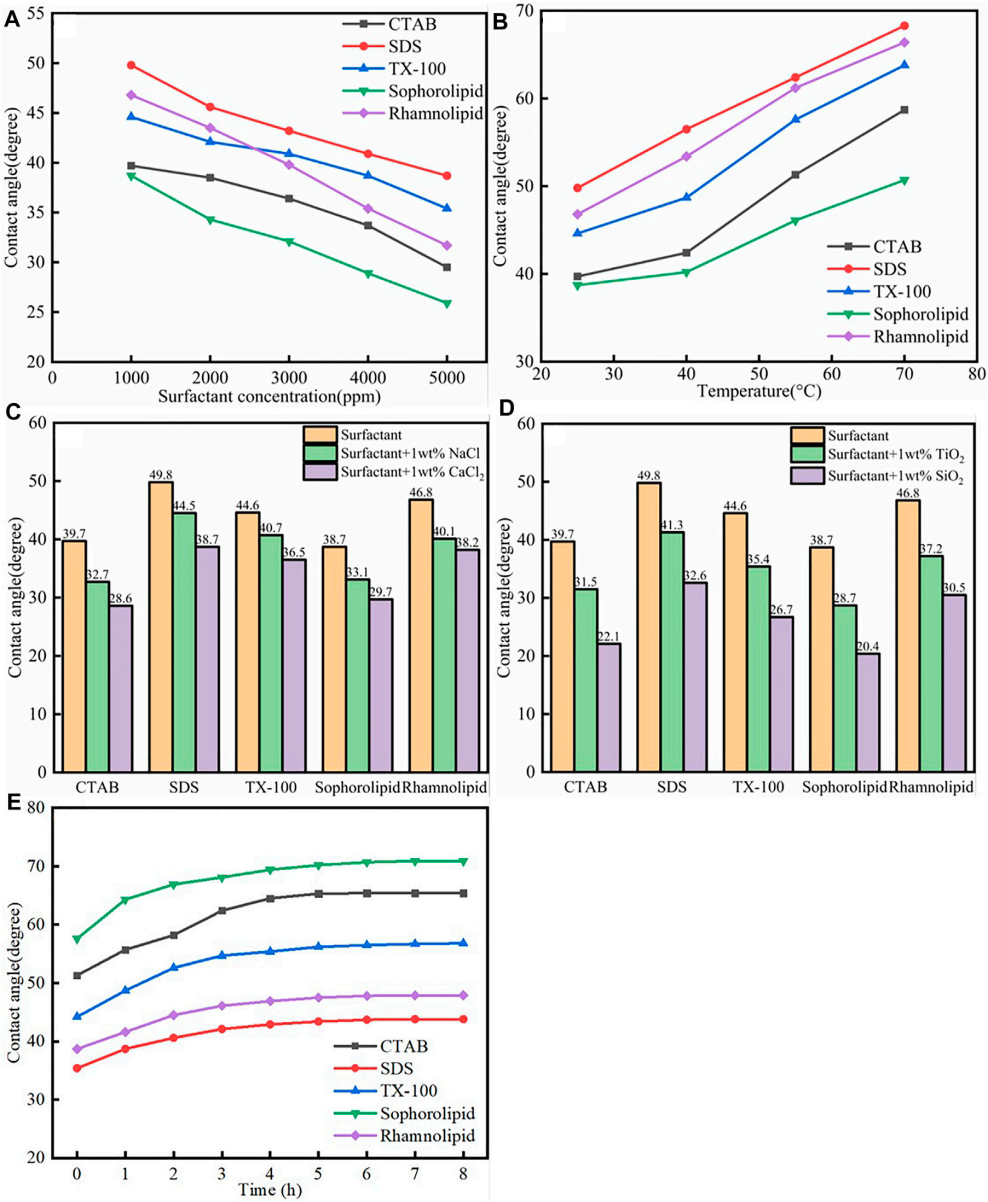
**(A)** Surfactants species; **(B)** Temperature; **(C)** Salinity; **(D)** Nanoparticles- assisted effect on the calcite surface wettability—static contact angle measurement. **(E)** The different surfactants assisted calcite surface wettability alteration-dynamic contact angle measurement.

The static and dynamic contact angle experiment device diagram was shown in Supplementary Figure S12. The static contact angle results were shown in Figure 11. As was shown in Figure 11, the five surfactants could make the calcite surface more hydrophilic. As was shown in Figure 11A, when the surfactants concentration increased, the contact angle on the surfactant altered calcite surface would decrease a lot, which means that the calcite surface become more hydrophilic. The reason was due to the fact that when surfactants concentration increased, the surfactants adsorption would increase, which made the calcite surface more hydrophilic. As was shown in Figure 11B, the contact angle would increase when the temperature increased. When the temperature increase, the surfactant adsorption would decrease, which made the calcite surface become less hydrophilic. As was shown in Figure 11C, the contact angle decreased with the high salinity. When the salinity increased, the surfactant adsorption would increase, which was beneficial to the calcite surface wettability alteration. As was shown in Figure 11D, the contact angle decreased when the surfactants work with SiO_2_/TiO_2_ nanoparticles, and the reason was because the SiO_2_/TiO_2_ nanoparticles would adsorb onto calcite surface, regardless of the surfactants adsorption. Therefore, although surfactants adsorption would decrease, the contact angle would still decrease.

The dynamic contact angle was shown in Figure 11E. As was shown in Figure 11E, the contact angle of the oil drops on the calcite surface (in aqueous solution) would increase with time passed, which means that the surface become more hydrophilic when the time passed. On the one hand, surfactants adsorption quantity would increase when the time passed. On the other hand, the surfactants could effective enhanced oil liberate.

### 5.2 Interaction Energy Analysis

**FIGURE 12:**
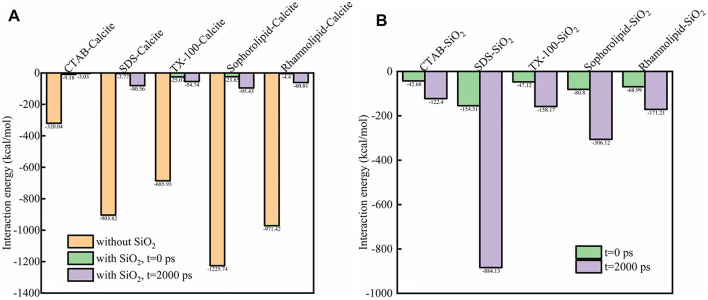
The interaction energy (kcal/mol) between **(A)** surfactant-calcite surface (with or without SiO_2_ nanoparticles); **(B)** surfactants-SiO_2_ nanoparticles.

Figure 12 showed the interaction energy among surfactants, SiO_2_ nanoparticles and calcite surface. As was shown in Figure 12, the interaction energy between CTAB, SDS, TX-100, sophorolipid and rhamnolipid with calcite surface were −320.04, −903.82, −685.93, −1,225.74, −971.42 kcal/mol, respectively. The interaction energy between biosurfactants (sophorolipid, rhamnolipid) and calcite surface was higher than that between chemical surfactants and calcite surface. The reason was due to the fact that the biosurfactants had high steric hindrance and molecular mass, which made them have strong interaction force with calcite surface. When the simulation time proceeds, the interaction energy of surfactants-calcite surface, surfactants-SiO_2_ nanoparticles would increase, which means that the surfactants adsorbed onto SiO_2_ nanoparticles surface.

### 5.3 Surface Tension Analysis

**FIGURE 13:**
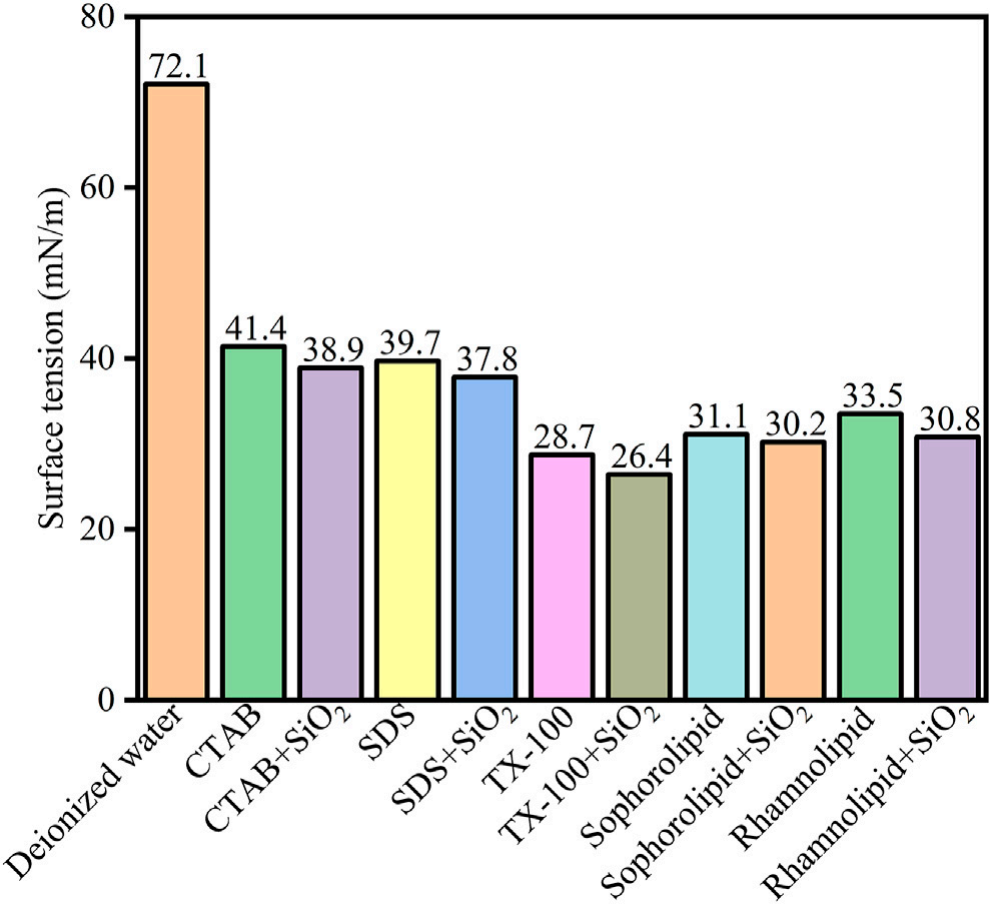
Surface tension (mN/m) of the deionized water, surfactants solutions (2000 ppm) and surfactant (2000 ppm)-SiO_2_ nanoparticles at 298 K.

The five surfactants and surfactants-SiO_2_ nanoparticles effect on surface tension was Figure 13. As was shown in Figure 13, the surfactants could effectively decrease surface tension. 2000 ppm CTAB, SDS, TX-100, sophorolipid and rhamnolipid could decrease the water surface tension from 72.1 mN/m to 41.4 mN/m, 39.7 mN/m, 28.7 mN/m, 31.1 mN/m, 33.5 mN/m, respectively. Besides, the surfactants-SiO_2_ nanoparticles could further decrease the water surface tension, and the corresponding surface tension was 38.9 mN/m, 37.8 mN/m, 26.4 mN/m, 30.2 mN/m, 30.8 mN/m, respectively. The results indicated that surfactants adsorbed onto the SiO_2_ nanoparticles surface, and then surfactants and SiO_2_ nanoparticles could synergistically decrease the water surface tension.

### 5.4 Similar Results From Experiment and Simulation

The experiment results could be verified by molecular dynamic simulation, and the detailed similar results from experiment and simulation was as follows.

1) The experiment results showed that the SiO_2_ nanoparticles could decrease the five surfactants adsorption onto the carbonate rocks, and the simulation results verified the results.
2) For the three chemical surfactants, the adsorption quantity order was CTAB > SDS > TX-100, and the simulation results were in accordance with the experiment results, the mean square displacement order of the chemical surfactants was as follows: CTAB > SDS > TX-100.
3) The contact angle measurements showed that the surfactants adsorption would make the carbonate surface more hydrophilic, and the simulation results verified the experiment results.

## 6 Conclusion

In this study, we studied the surfactants adsorption onto carbonate surface by experiment and molecular dynamics simulation, and the detailed conclusions were as follows:

1) CTAB, SDS, TX-100, sophorolipid and rhamnolipid adsorption onto carbonate rocks could be well fitted by Langmuir model, Freundlich model and Temkin model. Cationic surfactants adsorption quantity was higher than anionic surfactants, and the non-ionic surfactants adsorption quantity was the lowest.
2) When the temperature decreased or salinity increased, the surfactants adsorption would increase. Higher salinity could compress electric double layer which increased the surfactants adsorption. In addition, divalent ions (Ca^2+^) could make the surfactants adsorption quantity higher than monovalent ion (Na^+^).
3) TiO_2_ nanoparticles and SiO_2_ nanoparticles decreased the surfactants adsorption onto the carbonate rocks surface, and the reason was because the surfactants molecules adsorbed onto SiO_2_ nanoparticles surface, which increased the surfactants molecules steric hindrance. The contact angle measurement indicated that SiO_2_ nanoparticles adsorption could make the carbonate rock surface more hydrophilic.
4) The molecular dynamics simulation results showed that the surfactants molecules adsorbed onto the SiO_2_ nanoparticles surface, and the surfactants adsorption was decreased.

## Data Availability

The original contributions presented in the study are included in the article/Supplementary Material, further inquiries can be directed to the corresponding authors.
