# Supplementary material for: Study of the Adsorption Behavior of Surfactants on Carbonate Surface by Experiment and Molecular Dynamics Simulation
Source: Front Chem. 2022 Apr 7;10:847986. doi: 10.3389/fchem.2022.847986 (PMC9021538; doi:10.3389/fchem.2022.847986)
Supplement: Supplementary file 1 [file DataSheet1.docx]

**Study of the Adsorption Behavior of Surfactants on Carbonate Surface by Experiment and Molecular Dynamics Simulation**

Jinjian Hou^a, b, c^, Jinze Du ^a, b*^, Hong Sui^a,b,c*^, Shuanglong Lin^d^

*^a^ School of Chemical Engineering and Technology, Tianjin University, Tianjin 300072, China*

*^b^ National Engineering Research Centre of Distillation Technology, Tianjin 300072, China*

*^c^ Collaborative Innovation Center of Chemical Science and Engineering (Tianjin), 300072, China*

*^d^ School of chemical Engineering, Shijiazhuang University, Shijiazhuang 050035, China*

* Corresponding author: Jinze Du, Hong Sui

E-mail: [tjdujinze@hotmail.com](mailto:tjdujinze@hotmail.com) (Jinze Du), [suihong@tju.edu.cn](mailto:suihong@tju.edu.cn)(Hong Sui)

**Table S1.** Langmuir, Freundlich, Temkin and Linear model isotherm parameters of the five surfactants adsorption onto carbonate rock surface.

| Models | Parameters | CTAB | SDS | TX-100 | Sophorolipid | Rhamnolipid |
| --- | --- | --- | --- | --- | --- | --- |
| Langmuir | Q_m_(mg/g) | 28.12 | 24.81 | 13.45 | 7.18 | 43.57 |
|  | K_L_(L/mg) | 2.25×10^-4^ | 8.29×10^-5^ | 1.27×10^-4^ | 9.70×10^-5^ | 2.05×10^-4^ |
|  | R^2^ | 0.9720 | 0.9753 | 0.9775 | 0.9772 | 0.9666 |
| Freundlich | K_F_(L/mg) | 0.0813 | 0.0128 | 0.0122 | 0.0042 | 0.0880 |
|  | N | 1.502 | 1.185 | 1.294 | 1.227 | 1.455 |
|  | R^2^ | 0.9408 | 0.9647 | 0.9610 | 0.9656 | 0.9348 |
| Temkin | B | 9.015 | 6.818 | 3.470 | 1.7207 | 11.9857 |
|  | K_T_(L/mg) | 2.37×10^-3^ | 1.89×10^-3^ | 2.06×10^-3^ | 1.99×10^-3^ | 2.24×10^-3^ |
|  | R^2^ | 0.9920 | 0.9517 | 0.9658 | 0.9457 | 0.9896 |
| Linear | K_H_(L/mg) | 4.16×10^-3^ | 3.3×10^-3^ | 1.66×10^-3^ | 8.35×10^-4^ | 5.53×10^-3^ |
|  | R^2^ | 0.8901 | 0.9522 | 0.9378 | 0.9508 | 0.8869 |

**Table S2.** Langmuir, Freundlich, Temkin and Linear model isotherm parameters of the CTAB adsorption onto carbonate rock surface at different temperatures.

| Models | Parameters | 313 K | 328 K | 343 K |
| --- | --- | --- | --- | --- |
| Langmuir | Q_m_(mg/g) | 36.98 | 29.82 | 31.49 |
|  | K_L_(L/mg) | 1.98×10^-4^ | 2.32×10^-4^ | 1.94×10^-4^ |
|  | R^2^ | 0.9627 | 0.9445 | 0.9554 |
| Freundlich | K_F_(L/mg) | 0.0618 | 0.0701 | 0.0504 |
|  | N | 1.437 | 1.499 | 1.422 |
|  | R^2^ | 0.9319 | 0.9037 | 0.9233 |
| Temkin | B | 9.085 | 8.111 | 8.027 |
|  | K_T_(L/mg) | 2.21×10^-3^ | 2.24×10^-3^ | 2.13×10^-3^ |
|  | R^2^ | 0.9854 | 0.9823 | 0.9889 |
| Linear | K_H_(L/mg) | 4.2×10^-3^ | 3.68×10^-3^ | 3.69×10^-3^ |
|  | R^2^ | 0.8860 | 0.8449 | 0.8767 |

**Table S3.** Langmuir, Freundlich, Temkin and Linear model isotherm parameters of the SDS adsorption onto carbonate rock surface at different temperatures.

| Models | Parameters | 313 K | 328 K | 343 K |
| --- | --- | --- | --- | --- |
| Langmuir | Q_m_(mg/g) | 35.89 | 31.25 | 27.21 |
|  | K_L_(L/mg) | 6.76×10^-5^ | 7.20×10^-5^ | 6.29×10^-5^ |
|  | R^2^ | 0.9692 | 0.9640 | 0.9619 |
| Freundlich | K_F_(L/mg) | 9.44×10^-3^ | 9.17×10^-3^ | 7.10×10^-3^ |
|  | N | 1.139 | 1.145 | 1.118 |
|  | R^2^ | 0.9597 | 0.9532 | 0.9521 |
| Temkin | B | 6.876 | 6.477 | 6.092 |
|  | K_T_(L/mg) | 1.78×10^-3^ | 1.76×10^-3^ | 1.71×10^-3^ |
|  | R^2^ | 0.9505 | 0.9550 | 0.9559 |
| Linear | K_H_(L/mg) | 3.33×10^-3^ | 3.12×10^-3^ | 2.93×10^-3^ |
|  | R^2^ | 0.9507 | 0.9431 | 0.9446 |

**Table S4.** Langmuir, Freundlich, Temkin and Linear model isotherm parameters of the TX-100 adsorption onto carbonate rock surface at different temperatures.

| Models | Parameters | 313 K | 328 K | 343 K |
| --- | --- | --- | --- | --- |
| Langmuir | Q_m_(mg/g) | 14.54 | 15.18 | 15.92 |
|  | K_L_(L/mg) | 1.34×10^-4^ | 7.50×10^-4^ | 5.79×10^-5^ |
|  | R^2^ | 0.9677 | 0.9709 | 0.9742 |
| Freundlich | K_F_(L/mg) | 1.14×10^-2^ | 4.70×10^-3^ | 3.31×10^-3^ |
|  | N | 1.303 | 1.157 | 1.114 |
|  | R^2^ | 0.9475 | 0.9600 | 0.9663 |
| Temkin | B | 3.139 | 3.059 | 0.897 |
|  | K_T_(L/mg) | 2.03×10^-3^ | 1.80×10^-3^ | 9.91×10^-3^ |
|  | R^2^ | 0.9676 | 0.9578 | 0.9663 |
| Linear | K_H_(L/mg) | 1.49×10^-3^ | 1.48×10^-3^ | 1.39×10^-3^ |
|  | R^2^ | 0.9207 | 0.9491 | 0.9596 |

**Table S5.** Langmuir, Freundlich, Temkin and Linear model isotherm parameters of the sophorolipid adsorption onto carbonate rock surface at different temperatures.

| Models | Parameters | 313 K | 328 K | 343 K |
| --- | --- | --- | --- | --- |
| Langmuir | Q_m_(mg/g) | 8.08 | 7.82 | 5.51 |
|  | K_L_(L/mg) | 9.68×10^-5^ | 8.03×10^-5^ | 6.19×10^-5^ |
|  | R^2^ | 0.9733 | 0.9762 | 0.9614 |
| Freundlich | K_F_(L/mg) | 3.47×10^-3^ | 2.43×10^-3^ | 1.43×10^-3^ |
|  | N | 1.217 | 1.176 | 1.115 |
|  | R^2^ | 0.9600 | 0.9656 | 0.9518 |
| Temkin | B | 1.533 | 1.386 | 1.255 |
|  | K_T_(L/mg) | 1.92×10^-3^ | 1.85×10^-3^ | 1.70×10^-3^ |
|  | R^2^ | 0.9576 | 0.9573 | 0.9566 |
| Linear | K_H_(L/mg) | 7.38×10^-4^ | 6.70×10^-4^ | 6.04×10^-4^ |
|  | R^2^ | 0.9439 | 0.9535 | 0.9446 |

**Table S6.** Langmuir, Freundlich, Temkin and Linear model isotherm parameters of the rhamnolipid adsorption onto carbonate rock surface at different temperatures.

| Models | Parameters | 313 K | 328 K | 343 K |
| --- | --- | --- | --- | --- |
| Langmuir | Q_m_(mg/g) | 48.01 | 44.64 | 49.15 |
|  | K_L_(L/mg) | 1.95×10^-4^ | 1.88×10^-4^ | 1.41×10^-4^ |
|  | R^2^ | 0.9640 | 0.9544 | 0.9684 |
| Freundlich | K_F_(L/mg) | 7.77×10^-2^ | 6.72×10^-2^ | 4.08×10^-2^ |
|  | N | 1.431 | 1.409 | 1.311 |
|  | R^2^ | 0.9332 | 0.9221 | 0.9468 |
| Temkin | B | 11.76 | 11.37 | 10.97 |
|  | K_T_(L/mg) | 2.18×10^-3^ | 2.10×10^-3^ | 1.98×10^-3^ |
|  | R^2^ | 0.9895 | 0.9874 | 0.9890 |
| Linear | K_H_(L/mg) | 5.43×10^-3^ | 5.22×10^-3^ | 5.14×10^-3^ |
|  | R^2^ | 0.8874 | 0.8767 | 0.9166 |

**Table S7.** Langmuir, Freundlich, Temkin and Linear model isotherm parameters of five surfactants in 1 wt% NaCl solution adsorption onto carbonate rock surface.

| Models | Parameters | CTAB | SDS | TX-100 | Sophorolipid | Rhamnolipid |
| --- | --- | --- | --- | --- | --- | --- |
| Langmuir | Q_m_(mg/g) | 37.36 | 24.31 | 16.89 | 10.15 | 41.22 |
|  | K_L_(L/mg) | 3.45×10^-4^ | 1.72×10^-4^ | 1.41×10^-4^ | 2.44×10^-4^ | 2.58×10^-4^ |
|  | R^2^ | 0.9827 | 0.9808 | 0.9888 | 0.9936 | 0.9839 |
| Freundlich | K_F_(L/mg) | 0.1953 | 0.0409 | 0.0173 | 0.0275 | 0.1459 |
|  | N | 1.762 | 1.405 | 1.343 | 1.595 | 1.583 |
|  | R^2^ | 0.9439 | 0.9623 | 0.9755 | 0.9819 | 0.9535 |
| Temkin | B | 8.593 | 6.682 | 3.730 | 1.999 | 11.642 |
|  | K_T_(L/mg) | 3.08×10^-3^ | 2.3×10^-3^ | 2.24×10^-3^ | 2.94×10^-3^ | 2.62×10^-3^ |
|  | R^2^ | 0.9919 | 0.9726 | 0.9629 | 0.9670 | 0.9943 |
| Linear | K_H_(L/mg) | 3.93×10^-3^ | 3.17×10^-3^ | 1.8×10^-3^ | 9.60×10^-4^ | 5.39×10^-3^ |
|  | R^2^ | 0.8723 | 0.9306 | 0.9529 | 0.9488 | 0.8992 |

**Table S8.** Langmuir, Freundlich, Temkin and Linear model isotherm parameters of five surfactants in 1 wt% CaCl_2_ adsorption onto carbonate rock surface.

| Models | Parameters | CTAB | SDS | TX-100 | Sophorolipid | Rhamnolipid |
| --- | --- | --- | --- | --- | --- | --- |
| Langmuir | Q_m_(mg/g) | 40.18 | 27.61 | 19.45 | 10.01 | 46.32 |
|  | K_L_(L/mg) | 4.36×10^-4^ | 3.64×10^-4^ | 2.14×10^-4^ | 3.75×10^-4^ | 3.94×10^-4^ |
|  | R^2^ | 0.9929 | 0.9910 | 0.9866 | 0.9903 | 0.9947 |
| Freundlich | K_F_(L/mg) | 0.3644 | 0.1763 | 0.0354 | 0.0694 | 0.3546 |
|  | N | 1.952 | 1.832 | 1.500 | 1.860 | 1.894 |
|  | R^2^ | 0.9560 | 0.9684 | 0.9641 | 0.9702 | 0.9727 |
| Temkin | B | 9.349 | 6.108 | 3.855 | 2.207 | 10.339 |
|  | K_T_(L/mg) | 3.80×10^-3^ | 3.55×10^-3^ | 2.49×10^-3^ | 3.69×10^-3^ | 3.78×10^-3^ |
|  | R^2^ | 0.9937 | 0.9795 | 0.9844 | 0.9765 | 0.9848 |
| Linear | K_H_(L/mg) | 4.29×10^-3^ | 2.86×10^-3^ | 1.81×10^-3^ | 1.04×10^-3^ | 4.84×10^-3^ |
|  | R^2^ | 0.8773 | 0.9113 | 0.9229 | 0.9144 | 0.9125 |

**Table S9.** Langmuir, Freundlich, Temkin and Linear model isotherm parameters of the five surfactants-SiO_2_ nanoparticles adsorption onto carbonate rock surface.

| Models | Parameters | CTAB | SDS | TX-100 | Sophorolipid | Rhamnolipid |
| --- | --- | --- | --- | --- | --- | --- |
| Langmuir | Q_m_(mg/g) | 39.57 | 38.16 | 21.87 | 9.72 | 46.18 |
|  | K_L_(L/mg) | 2.06×10^-4^ | 6.27×10^-5^ | 7.31×10^-5^ | 3.86×10^-5^ | 1.66×10^-4^ |
|  | R^2^ | 0.9706 | 0.9776 | 0.9842 | 0.9813 | 0.9554 |
| Freundlich | K_F_(L/mg) | 6.12×10^-2^ | 8.52×10^-3^ | 5.7×10^-3^ | 1.36×10^-3^ | 5.33×10^-2^ |
|  | N | 1.461 | 1.135 | 1.168 | 1.072 | 1.358 |
|  | R^2^ | 0.9434 | 0.9699 | 0.9762 | 0.9771 | 0.9271 |
| Temkin | B | 8.045 | 6.294 | 3.353 | 1.578 | 11.518 |
|  | K_T_(L/mg) | 2.28×10^-3^ | 1.82×10^-3^ | 1.89×10^-3^ | 1.74×10^-3^ | 2.01×10^-3^ |
|  | R^2^ | 0.99342 | 0.9431 | 0.94978 | 0.93573 | 0.99073 |
| Linear | K_H_(L/mg) | 0.00373 | 0.00307 | 0.00164 | 7.78061 | 0.00531 |
|  | R^2^ | 0.89779 | 0.96213 | 0.9665 | 0.97418 | 0.88811 |

**Table S10.** Langmuir, Freundlich, Temkin and Linear model isotherm parameters of the five surfactants-TiO_2_ nanoparticles adsorption onto carbonate rock surface.

| Models | Parameters | CTAB | SDS | TX-100 | Sophorolipid | Rhamnolipid |
| --- | --- | --- | --- | --- | --- | --- |
| Langmuir | Q_m_(mg/g) | 33.67 | 34.75 | 20.13 | 4.78 | 46.68 |
|  | K_L_(L/mg) | 1.88×10^-4^ | 4.63×10^-5^ | 7.19×10^-5^ | 3.45×10^-5^ | 1.45×10^-4^ |
|  | R^2^ | 0.9704 | 0.9776 | 0.9689 | 0.9810 | 0.9580 |
| Freundlich | K_F_(L/mg) | 5.01×10^-2^ | 5.81×10^-3^ | 3.25×10^-3^ | 6.64×10^-4^ | 4.12×10^-2^ |
|  | N | 1.420 | 1.0891 | 1.1471 | 0.994 | 1.311 |
|  | R^2^ | 0.9450 | 0.9719 | 0.9590 | 0.9803 | 0.9339 |
| Temkin | B | 7.893 | 5.956 | 2.269 | 1.460 | 11.211 |
|  | K_T_(L/mg) | 2.2×10^-3^ | 1.74×10^-3^ | 1.76×10^-3^ | 1.61×10^-3^ | 1.94×10^-3^ |
|  | R^2^ | 0.9917 | 0.9412 | 0.9645 | 0.9324 | 0.9908 |
| Linear | K_H_(L/mg) | 3.67×10^-3^ | 0.00292 | 0.00109 | 7.23×10^-4^ | 5.21×10^-3^ |
|  | R^2^ | 0.9038 | 0.9677 | 0.9487 | 0.9825 | 0.9016 |

**Table S11**. The different surfactants system energy change of with time.

| Surfac-tants | Energy | 0 ps | 500 ps | 1000 ps | 1500 ps | 1600 ps | 1700 ps | 1800 ps | 1900 ps | 2000 ps |
| --- | --- | --- | --- | --- | --- | --- | --- | --- | --- | --- |
| CTAB | Potential energy (kcal/mol) | -482.9 | -830.6 | -862.5 | -897.8 | -894.4 | -907.8 | -885.4 | -914.1 | -866.7 |
|  | Kinetic energy (kcal/mol) | 1142.4 | 1076.1 | 1110.1 | 1124.4 | 1116.3 | 1139.1 | 1123.6 | 1120.9 | 1090.9 |
|  | Non-bond energy(kcal/mol) | -486.5 | -916.8 | -897.2 | -918.5 | -921.3 | -913.9 | -919.1 | -931.2 | -933.8 |
|  | Total energy(kcal/mol) | 659.5 | 241.0 | 247.6 | 226.6 | 221.9 | 231.4 | 238.3 | 206.8 | 224.2 |
| SDS | Potential energy (kcal/mol) | -4811.8 | -5634.3 | -5659.8 | -5654.4 | -5646.5 | -5659.2 | -5649.0 | -5660.0 | -5687.8 |
|  | Kinetic energy (kcal/mol) | 801.1 | 769.6 | 772.9 | 768.1 | 766.9 | 776.2 | 774.7 | 760.5 | 792.6 |
|  | Non-bond energy(kcal/mol) | -4691.9 | -5555.3 | -5582.8 | -5593.9 | -5591.2 | -5604.4 | -5579.7 | -5604.0 | -5590.5 |
|  | Total energy(kcal/mol) | -4010.6 | -4864.7 | -4886.9 | -4886.2 | -4879.5 | -4883.0 | -4874.3 | -4899.5 | -4895.2 |
| TX-  100 | Potential energy (kcal/mol) | 230.0 | -323.2 | -325.3 | -330.5 | -320.6 | -336.2 | -328.8 | -388.8 | -349.2 |
|  | Kinetic energy (kcal/mol) | 894.5 | 902.4 | 910.4 | 919.4 | 884.6 | 910.8 | 895.8 | 944.7 | 903.2 |
|  | Non-bond energy(kcal/mol) | -210.8 | -758.8 | -771.5 | -743.3 | -766.2 | -787.7 | -805.1 | -788.2 | -816.0 |
|  | Total energy(kcal/mol) | 1124.5 | 579.1 | 585.2 | 588.8 | 564.0 | 574.6 | 567.0 | 555.9 | 554.0 |
| Sophor-olipid | Potential energy (kcal/mol) | 1219.2 | 29.7 | 11.8 | -71.5 | -38.9 | -62.3 | -110.4 | -72.4 | -67.0 |
|  | Kinetic energy (kcal/mol) | 1830.2 | 1859.0 | 1852.1 | 1879.8 | 1838.8 | 1856.3 | 1857.8 | 1833.5 | 1823.5 |
|  | Non-bond energy(kcal/mol) | 778.6 | -422.0 | -450.0 | -494.8 | -552.7 | -548.7 | -584.0 | -536.3 | -590.9 |
|  | Total energy(kcal/mol) | 3049.4 | 1888.7 | 1863.8 | 1808.3 | 1780.0 | 1794.0 | 1747.4 | 1761.1 | 1756.4 |
| Rhamn-olipid | Potential energy (kcal/mol) | -167.3 | -855.1 | -853.2 | -916.2 | -876.2 | -902.4 | -919.5 | -900.7 | -901.6 |
|  | Kinetic energy (kcal/mol) | 979.6 | 941.7 | 926.6 | 968.3 | 925.6 | 935.9 | 960.1 | 940.0 | 931.5 |
|  | Non-bond energy(kcal/mol) | -251.8 | -1053.7 | -1105.9 | -1113.5 | -1133.9 | -1119.2 | -1144.9 | -1107.0 | -1126.0 |
|  | Total energy (kcal/mol) | 812.3 | 86.6 | 73.4 | 52.0 | 49.4 | 33.5 | 40.6 | 39.3 | 29.9 |

**Table S12**. The different surfactants-SiO_2_ nanoparticles system energy change of with time.

|  | Simulation time | 0ps | 500ps | 1000 ps | 1500 ps | 1600 ps | 1700 ps | 1800 ps | 1900 ps | 2000 ps |
| --- | --- | --- | --- | --- | --- | --- | --- | --- | --- | --- |
| CTAB-SiO_2_ | Potential energy (kcal/mol) | -22955.4 | -25960.2 | -25872.4 | -25974.0 | -25944.0 | -25949.9 | -25949.9 | -25916.7 | -25850.0 |
|  | Kinetic energy (kcal/mol) | 3985.1 | 3979.8 | 3892.2 | 3931.5 | 3914.7 | 3923.0 | 3996.0 | 3883.4 | 3890.2 |
|  | Non-bond energy(kcal/mol) | -25166.9 | -28261.1 | -28137.6 | -28283.6 | -28264.6 | -28224.8 | -28186.8 | -28212.6 | -28273.6 |
|  | Total energy(kcal/mol) | -18970.4 | -21980.4 | -21980.2 | -22042.4 | -22029.3 | -22026.9 | -21953.4 | -22033.3 | -21959.8 |
| SDS-SiO_2_ | Potential energy (kcal/mol) | -25179.6 | -27952.6 | -28076.2 | -28175.2 | -28133.4 | -28165.7 | -28146.8 | -28162.2 | -28236.9 |
|  | Kinetic energy (kcal/mol) | 3731.8 | 3749.0 | 3751.8 | 3782.4 | 3759.9 | 3770.4 | 3720.3 | 3722.7 | 3794.0 |
|  | Non-bond energy(kcal/mol) | -27312.5 | -30101.9 | -30268.8 | -30397.8 | -30309.3 | -30386.7 | -30399.0 | -30395.6 | -30400.6 |
|  | Total energy(kcal/mol) | -21447.8 | -24203.6 | -24324.4 | -24392.8 | -24373.5 | -24395.3 | -24426.5 | -24439.6 | -24442.9 |
| TX-  100-  SiO_2_ | Potential energy (kcal/mol) | -22413.6 | -25101.1 | -25287.9 | -25380.3 | -25357.9 | -25356.6 | -25447.5 | -25410.0 | -25522.7 |
|  | Kinetic energy (kcal/mol) | 3811.4 | 3824.3 | 3793.1 | 3822.0 | 3816.5 | 3781.2 | 3815.8 | 3862.6 | 3812.7 |
|  | Non-bond energy(kcal/mol) | -24810.0 | -27550.3 | -27763.9 | -27767.9 | -27802.9 | -27853.4 | -2789.8 | -27808.2 | -28007.8 |
|  | Total energy(kcal/mol) | -18602.2 | -21276.8 | -21494.8 | -21558.3 | -21541.4 | -21575.5 | -21631.7 | -21627.4 | -21710.0 |
| Sophor-olipid-SiO_2_ | Potential energy (kcal/mol) | -21999.8 | -24811.4 | -24969.5 | -25005.0 | -25058.0 | -25055.5 | -25129.8 | -25060.6 | -25104.6 |
|  | Kinetic energy (kcal/mol) | 4307.8 | 4280.9 | 4318.7 | 4262.1 | 4297.7 | 4287.0 | 4302.9 | 4274.6 | 4330.9 |
|  | Non-bond energy(kcal/mol) | -24337.3 | -27252.0 | -27378.0 | -27476.3 | -27475.0 | -27492.3 | -27498.5 | -27482.2 | -27552.5 |
|  | Total energy(kcal/mol) | -17692.0 | -20530.5 | -20650.8 | -20742.9 | -20760.3 | -20768.5 | -20828.9 | -20786.0 | -20773.7 |
| Rhamn-olipid-SiO_2_ | Potential energy (kcal/mol) | -22716.5 | -25455.0 | -25490.2 | -25668.2 | -25641.3 | -25716.4 | -25635.0 | -25645.1 | -25714.3 |
|  | Kinetic energy (kcal/mol) | 3802.4 | 3846.5 | 3823.0 | 3886.6 | 3877.2 | 3865.3 | 3820.1 | 3818.7 | 3799.3 |
|  | Non-bond energy(kcal/mol) | -24963.1 | -27709.6 | -27756.8 | -27925.4 | -27925.6 | -27983.3 | -28022.0 | -28005.2 | -28057.0 |
|  | Potential energy (kcal/mol) | -18914.0 | -21608.5 | -21667.2 | -21781.6 | -21764.1 | -21851.1 | -21815.0 | -21826.4 | -21914.9 |

**Table S13**. The different surfactants-SiO_2_ system temperature (K) change of with time.

| Surfactants | Temperature  (K) | 0  ps | 500  ps | 1000 ps | 1500 ps | 1600 ps | 1700 ps | 1800 ps | 1900 ps | 2000 ps |
| --- | --- | --- | --- | --- | --- | --- | --- | --- | --- | --- |
| CTAB | Without SiO_2_ | 304.2 | 286.5 | 295.6 | 299.4 | 308.5 | 303.3 | 299.2 | 298.4 | 290.5 |
|  | With SiO_2_ | 303.2 | 302.8 | 296.1 | 299.1 | 297.8 | 298.4 | 304.0 | 295.4 | 295.9 |
| SDS | Without SiO_2_ | 312.5 | 300.2 | 301.5 | 299.7 | 299.2 | 302.8 | 302.2 | 296.7 | 309.2 |
|  | With SiO_2_ | 297.4 | 298.7 | 299.0 | 301.4 | 299.6 | 300.4 | 296.5 | 296.6 | 302.3 |
| TX-100 | Without SiO_2_ | 294.2 | 296.8 | 299.4 | 302.4 | 290.9 | 299.6 | 294.6 | 310.7 | 297.1 |
|  | With SiO_2_ | 298.1 | 299.1 | 296.6 | 298.9 | 298.5 | 295.7 | 298.4 | 302.1 | 298.2 |
| Sophorolipid | Without SiO_2_ | 298.1 | 302.7 | 301.6 | 306.1 | 299.5 | 302.3 | 302.5 | 298.6 | 297.0 |
|  | With SiO_2_ | 300.5 | 298.6 | 301.2 | 297.3 | 299.8 | 299.0 | 300.1 | 298.1 | 302.1 |
| Rhamnolipid | Without SiO_2_ | 310.0 | 298.0 | 293.2 | 306.4 | 293.0 | 296.2 | 303.8 | 297.5 | 294.8 |
|  | With SiO_2_ | 296.0 | 299.4 | 297.6 | 302.5 | 301.8 | 300.9 | 297.3 | 297.2 | 295.7 |


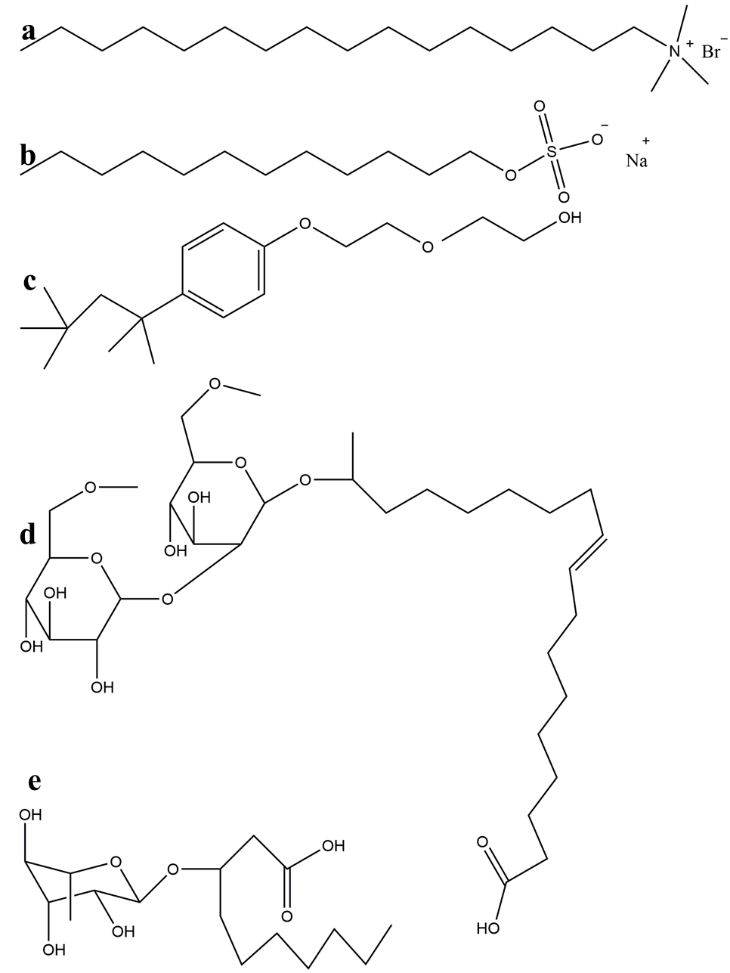


**Figure S1**. The structure of five surfactants (a)CTAB；(b)SDS; (c) TX-100;

(d) sophorolipid; (e) rhamnolipid


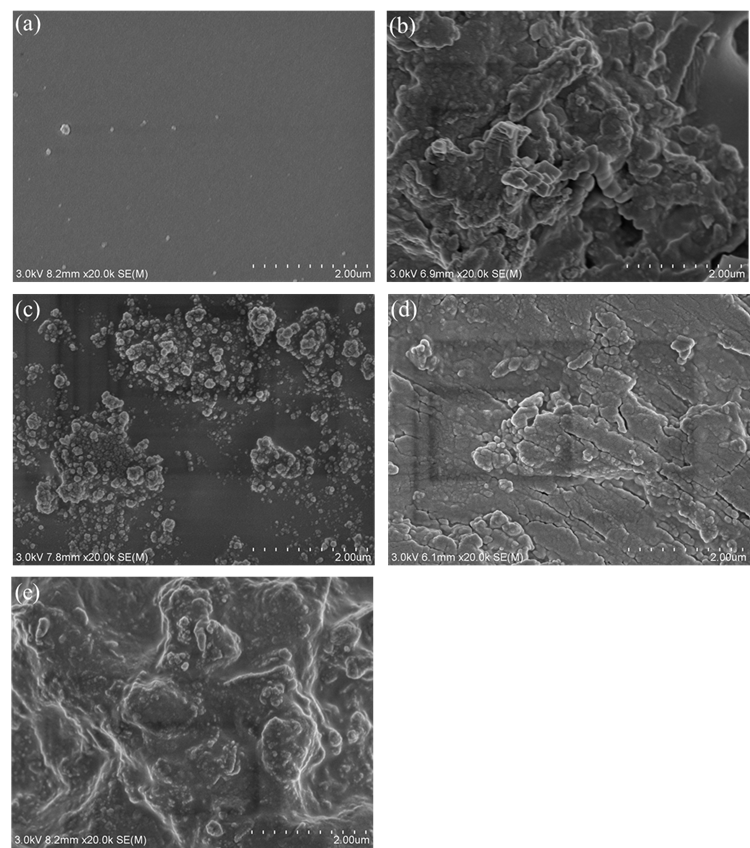


**Figure S2.** SEM figures of (a) CTAB; (b) SDS; (c) TX-100; (d) rhamnolipid; (e) sophorolipid adsorption onto the carbonate rock surface.


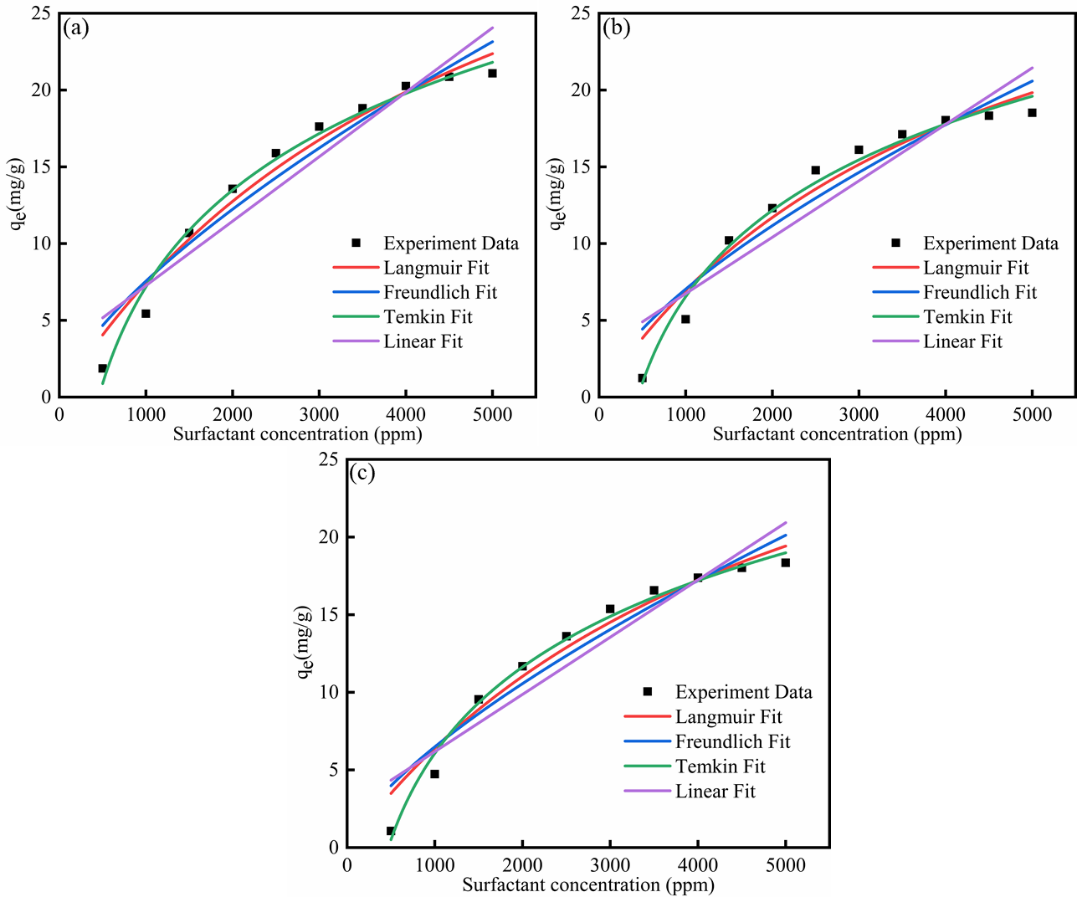


**Figure S3**. Different isotherm models fitting for CTAB adsorption onto carbonate rock at (a) 313 K, (b) 328 K, (c) 343 K.

**
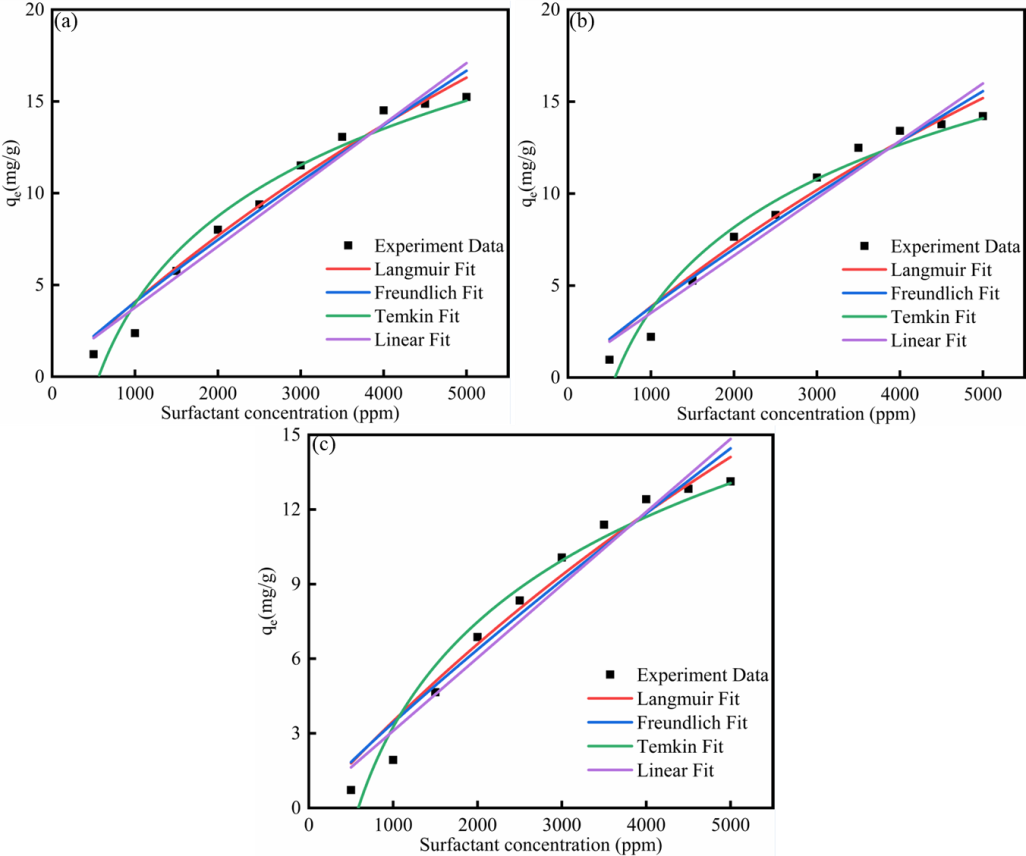
**

**Figure S4.** Different isotherm models fitting for SDS adsorption onto carbonate rock at (a) 313 K, (b) 328 K, (c) 343K.

**
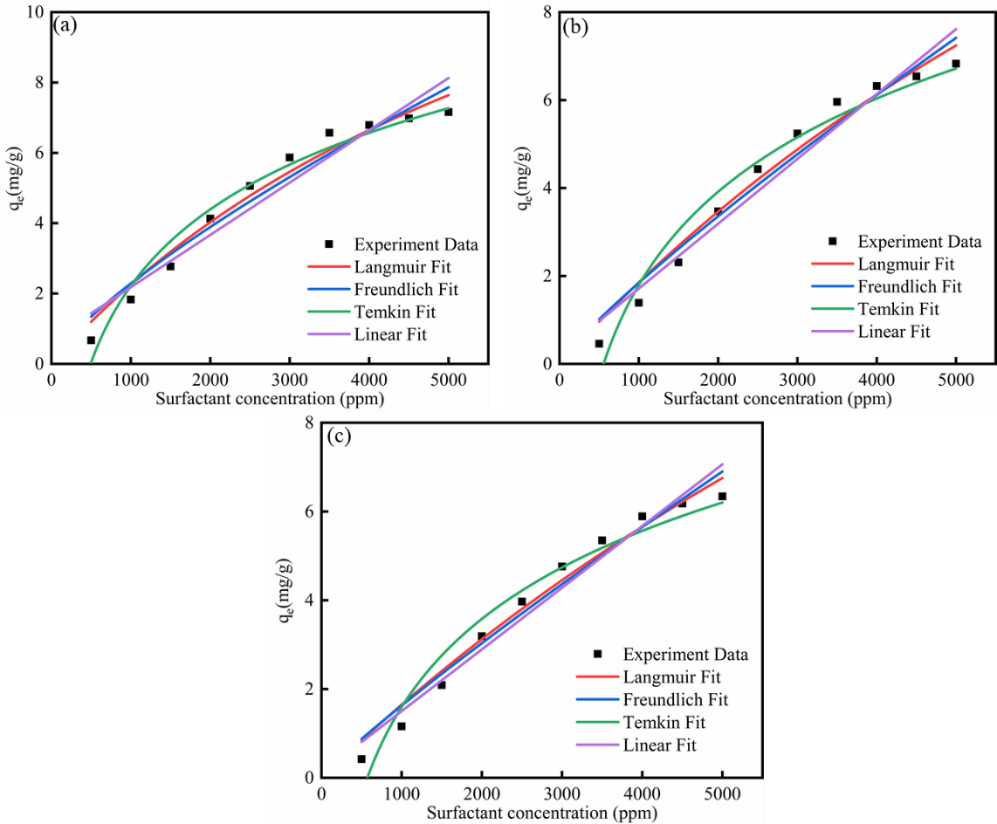
**

**Figure S5.** Different isotherm models fitting for TX-100 adsorption onto carbonate rock at (a) 313 K, (b) 328 K, (c) 343K.

**
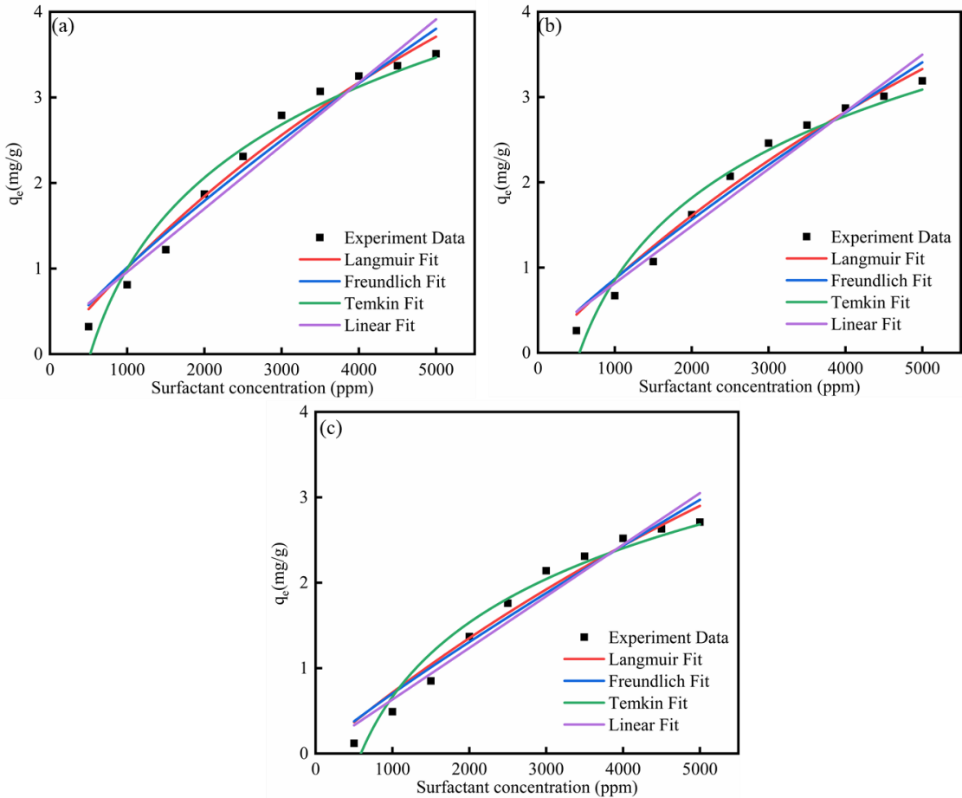
**

**Figure S6.** Different isotherm models fitting for sophorolipid adsorption onto carbonate rock at (a) 313 K, (b) 328 K, (c) 343K.

**
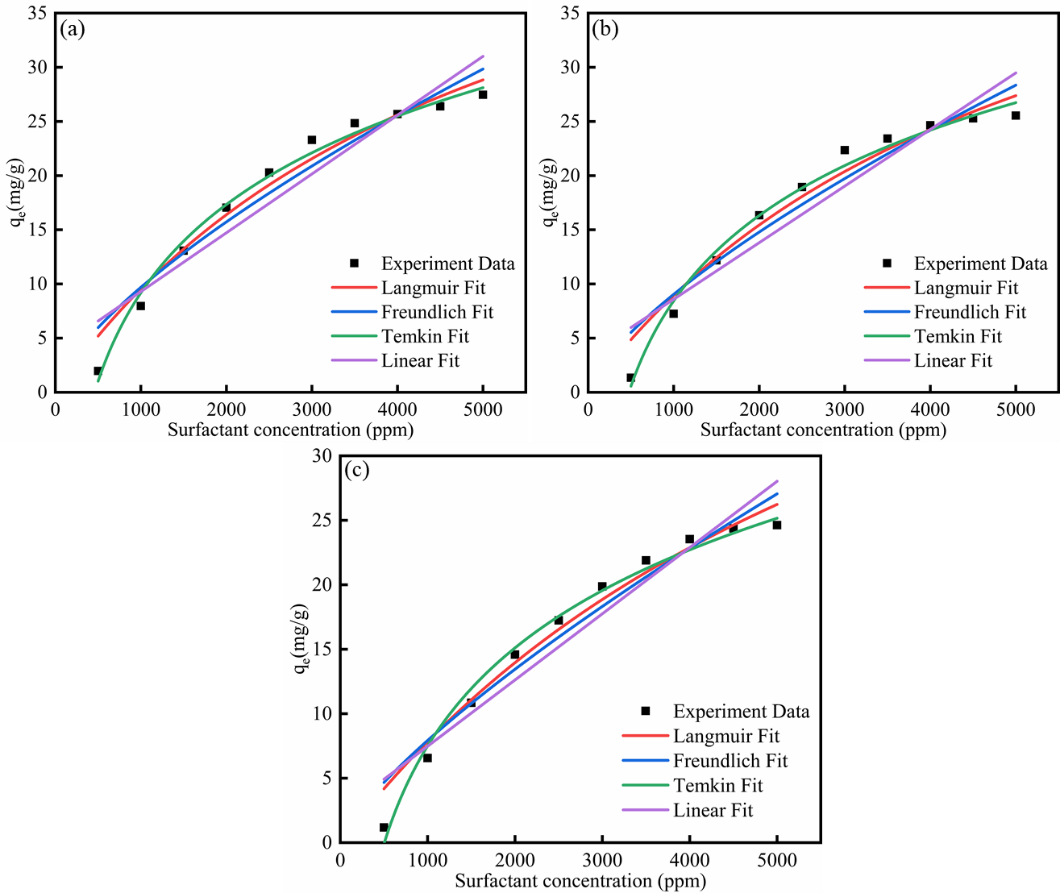
**

**Figure S7.** Different isotherm models for rhamnolipid adsorption onto carbonate rock at (a) 313 K, (b) 328 K, (c) 343K.

**
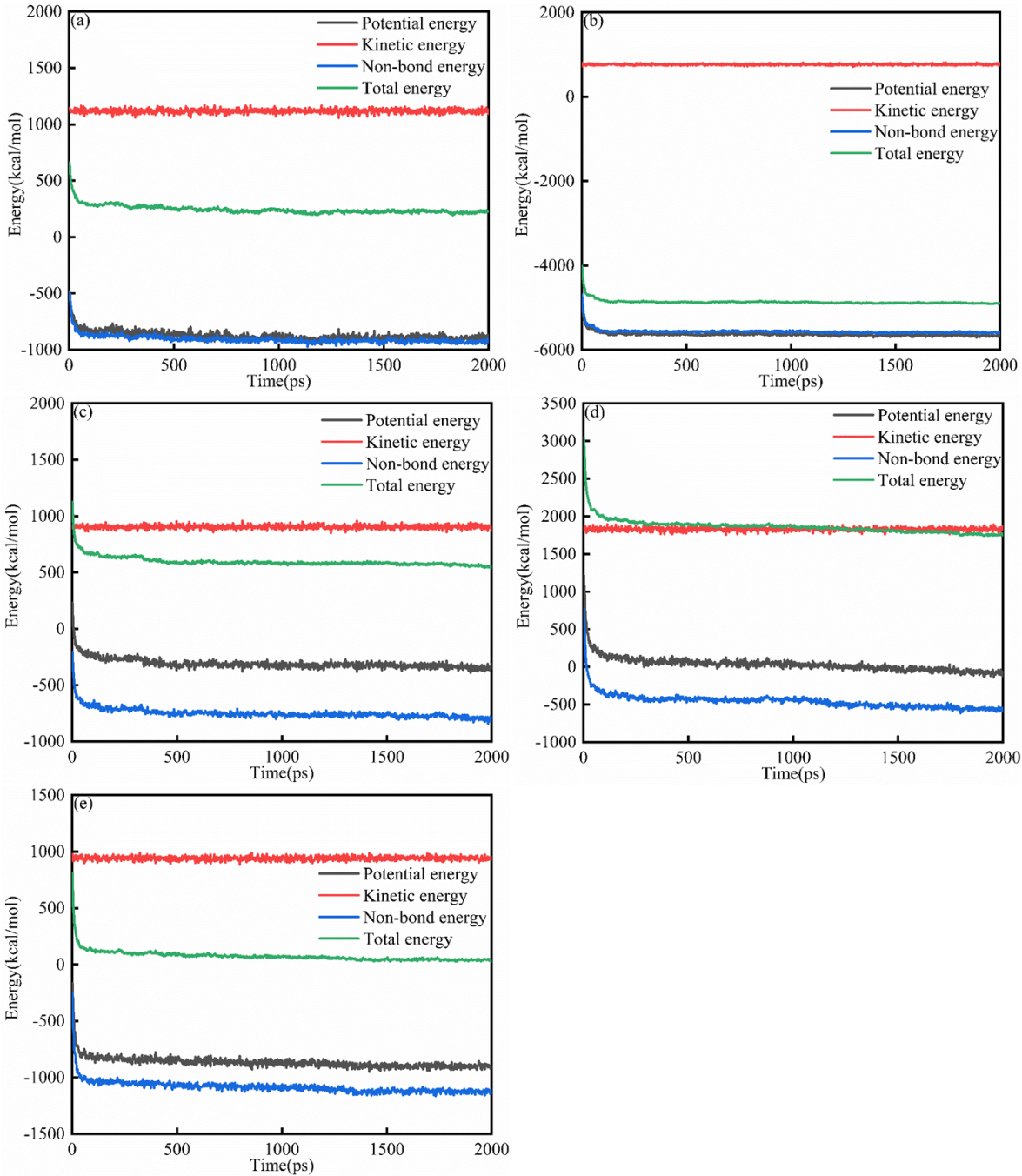
**

**Figure S8.** The different surfactants (a) CTAB; (b) SDS; (c) TX-100; (d) Sophorolipid; (e) Rhamnolipid system energy change of with time.

**
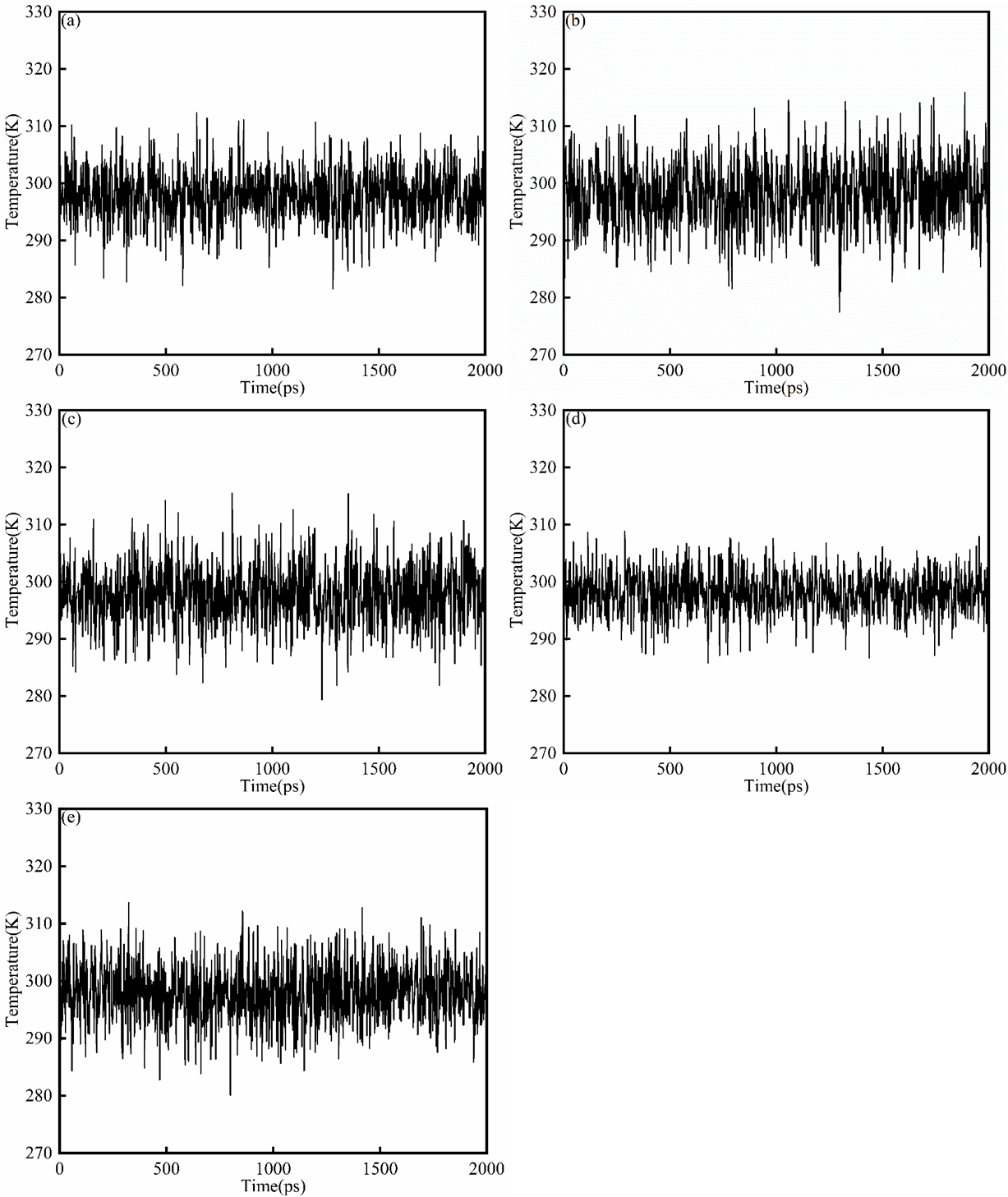
**

**Figure S9.** The different surfactants (a) CTAB; (b) SDS; (c) TX-100; (d) Sophorolipid; (e) Rhamnolipid system temperature change of with time.

**
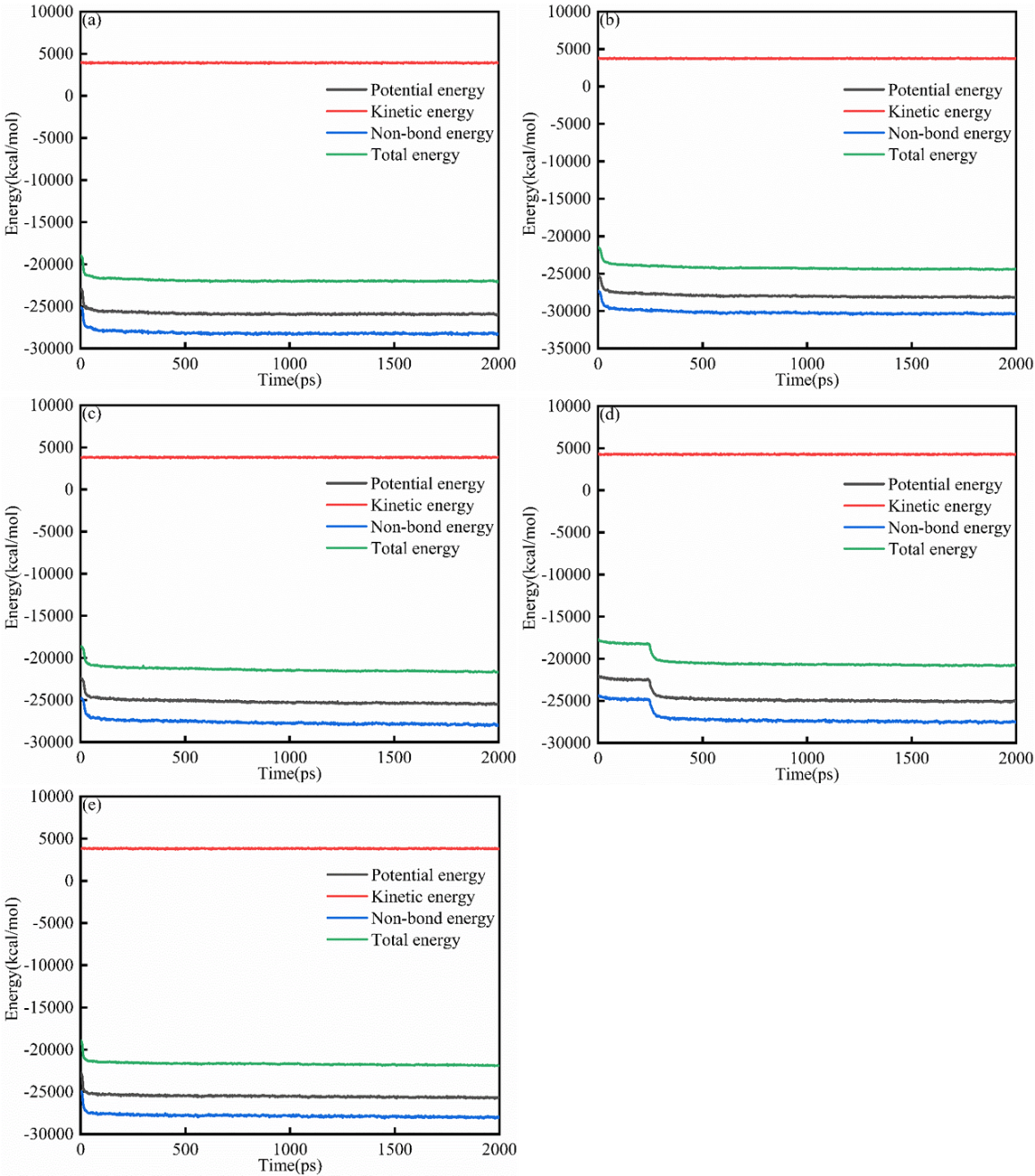
**

**Figure S10.** The different surfactants (a) CTAB-SiO_2_; (b) SDS-SiO_2_;

(c) TX-100-SiO_2_; (d) Sophorolipid-SiO_2_; (e) Rhamnolipid-SiO_2_ system energy change of with time.

**
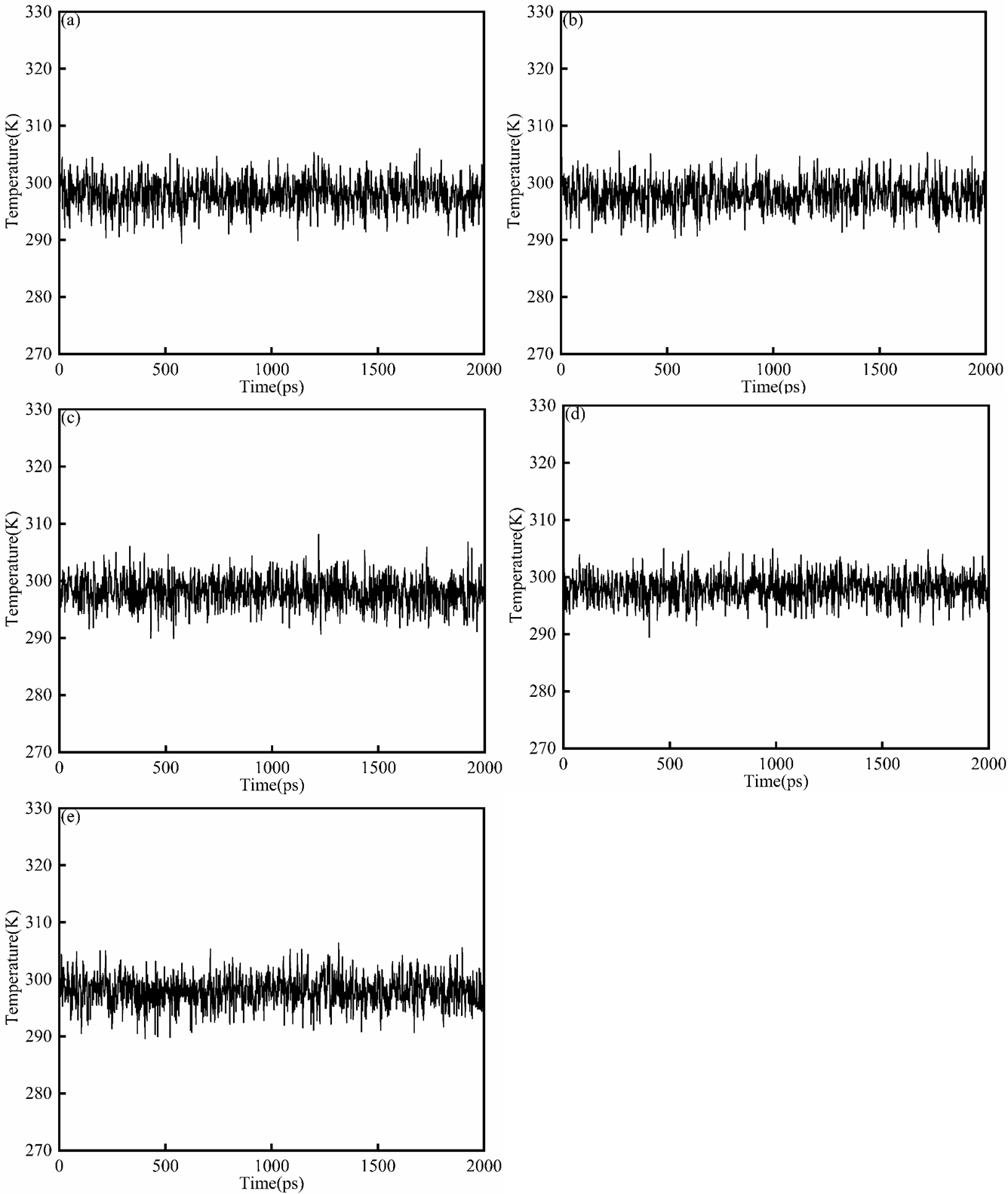
**

**Figure S11.** The different surfactants (a) CTAB-SiO_2_; (b) SDS-SiO_2_; (c) TX-100-SiO_2_; (d) Sophorolipid-SiO_2_; (e) Rhamnolipid-SiO_2_ system temperature change of with time.


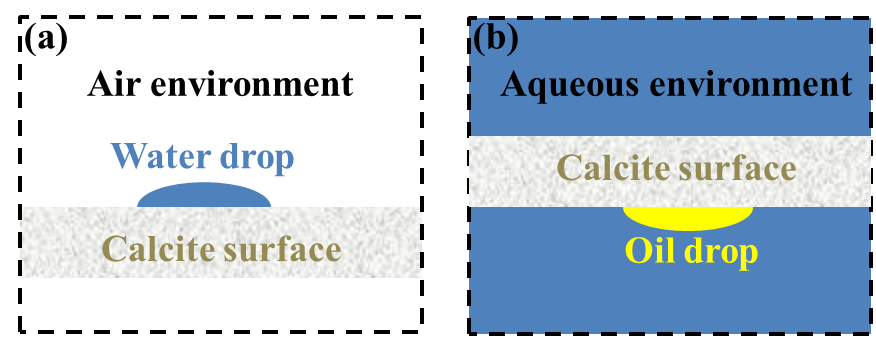


**Figure S12. (a)** Static; **(b)** Dynamic contact angle experiment device diagram
